# Meta-Analysis of Laparoscopic versus Open Hepatectomy for Live Liver Donors

**DOI:** 10.1371/journal.pone.0165319

**Published:** 2016-10-27

**Authors:** Jun Xu, Chen Hu, Hua-Li Cao, Mang-Li Zhang, Song Ye, Shu-Sen Zheng, Wei-Lin Wang

**Affiliations:** 1 Department of Hepatobiliary and Pancreatic Surgery, First Affiliated Hospital, Zhejiang University School of Medicine, Hangzhou, China; 2 Key Laboratory of Combined Multi-organ Transplantation, Ministry of Public Health, Hangzhou, China; 3 Department of Dermatology, Second Affiliated Hospital, Zhejiang University School of Medicine, Hangzhou, China; Istituto Mediterraneo per i Trapianti e Terapie ad Alta Specializzazione, ITALY

## Abstract

**Objective:**

To document the safety and efficacy of laparoscopic living donor hepatectomy in comparison with open liver resection for living donor liver transplantation.

**Methods:**

Medline database, EMASE and Cochrane library were searched for original studies comparing laparoscopic living donor hepatectomy (LLDH) and open living donor hepatectomy (OLDH) by January 2015. Meta-analysis was performed to evaluate donors’ perioperative outcomes.

**Results:**

Nine studies met selection criteria, involving 1346 donors of whom 318 underwent LLDH and 1028 underwent OLDH. The Meta analysis demonstrated that LLDH group had less operative blood loss [patients 1346; WMD: -56.09 mL; 95%CI: -100.28-(-11.90) mL; *P* = 0.01], shorter hospital stay [patients 737; WMD: -1.75 d; 95%CI: -3.01-(-0.48) d; *P* = 0.007] but longer operative time (patients 1346; WMD: 41.05 min; 95%CI: 1.91–80.19 min; *P* = 0.04), compared with OLDH group. There were no significant difference in other outcomes between LLDH and OLDH groups, including overall complication, bile leakage, postoperative bleeding, pulmonary complication, wound complication, time to dietary intake and period of analgesic use.

**Conclusions:**

LLDH appears to be a safe and effective option for LDLT. It improves donors’ perioperative outcomes as compared with OLDH.

## Introduction

Living donor liver transplantation(LDLT) has developed rapidly over the couple decades since the first treatment in 1989 in children[1]. The indication for LDLT has been extended to adult recipients[2–4]. LDLT serves as an established treatment for patients with end-stage liver disease (ESLD) when a deceased donor liver is not available. Partial hepatic allografts from live donors, compared with deceased donors, have been found to reduce the risk of the recipient dying on the waiting list. And the recipient survival is comparable to that in cadaveric liver transplantation[5–9].

The major concerns of potential donor are mainly the pain, morbidity associated with surgery and postoperative recovery. The method of open living donor hepatectomy (OLDH) has been nowadays challenged by less invasive techniques, although the most majority of procedures are still conventional open donor partial hepatectomy. Laparoscopic living donor hepatectomy (LLDH) are being increasingly performed in experienced centers[10].

The first LLDH was performed for a pediatric recipient by Cherqui in 2002 [11]. Since then, many transplant centers worldwide have adopted LLDH. Smaller incision sizes have stimulated reports of many investigations of lower blood loss, shorter hospital stay and faster physical recovery[12, 13]. However, the LLDH still remains the most controversial application of laparoscopic liver surgery[14]. The overriding concern for the transplant community is donor safety. Advocates of OLDH have concerned that laparoscopic surgery has been too rapidly extended to living liver donor candidates[15].

Several studies have compared donors’ perioperative complications between LLDH and the widely used standard OLDH. However, no definite consensus has been reached. We therefore performed a systematic review and meta-analysis to better clarify this issue.

## Materials and Methods

### Study Selection

We attempted to report this meta-analysis follow the proposed MOOSE (meta-analysis of observational studies in epidemiology) guidelines[16]. The MEDLINE, EMBASE and Cochrane database were searched to retrieve relevant studies published from January 2002 to January 2015. The following medical search headings and keywords were used: “laparoscopy” or “laparoscopic” or “minimally invasive surgery” or “minimal access surgery” and “hepatectomy” or “liver resection” or “hepatic resection” or “liver segmentectomy” or “hepatic segmentectomy” and “living donor” or “liver donor”. The “related articles” function was used to broaden our search. Reference lists of all retrieved articles were manually searched for additional studies. No language restriction was applied.

### Data Extraction

Data extraction was carried out by two independent reviewers (J.X. and C.H.). The following parameters were extracted: study name, first authors, publication year, study population characteristics, study design, number of subjects operated on with each procedure, and outcomes of interest. Disagreement between the reviewers were resolved by discussion and consensus between all authors.

### Outcomes of Interest

Operative outcomes: Operative time and intraoperative blood loss.

Postoperative outcomes: overall complication, bile leakage, postoperative bleeding, pulmonary complication, wound complication, period of analgesic use, time to dietary intake and hospital stay.

### Criteria for Inclusion and Exclusion

To be selected, a study had to meet the following criteria: (1) looked at laparoscopic and open donor hepatectomy surgical techniques for live liver donors. (2) compared the perioperative complications for LLDH and OLDH. (3) reported on at least one of the outcomes mentioned and had detailed the demographics to enable comparison and stratification of outcomes.

Abstracts, letters, editorials, expert opinions, case reports, reviews without original data and studies lacking control groups were excluded. Studies with less complete date from the same author or institution that contained significant overlap of patient data were also excluded.

### Qualitative Analysis

The quality of studies was assessed according to the Newcastle-Ottawa Scale (NOS)[17], by examining three factors: patient selection, comparability of the study groups and assessment of outcome. Studies achieving more than 6 points (maximum 11) were defined as higher quality.

### Statistical Methods

The meta-analysis was performed using the Review Manager version 5.3 software (The Cochrane Collaboration, Oxford, United Kingdom). Analyses were performed using odds ratios (OR) for dichotomous variables and weighted mean differences (WMD) for continuous variables. The corresponding 95% confidence intervals (CIs) were reported. Heterogeneity was assessed by using the inconsistency statistic (I^2^)[18]. The fixed-effects model was used for calculations of all outcomes. However, when the heterogeneity was more than 25%, a random-effects model was used[19]. Statistical significance was considered at *P* < 0.05. Sensitivity analysis was performed by removing extreme data (the maximum or the minimum) and analyzing the effect on the overall results. Graphical funnel plots were generated to make visual inspections for publication bias. Egger test (Stata version 12.0) was used to detect the funnel plot asymmetry statistically[20]. Publication bias was considered at *P* < 0.05.

## Results

### Flow of Included Studies

**Fig 1:**
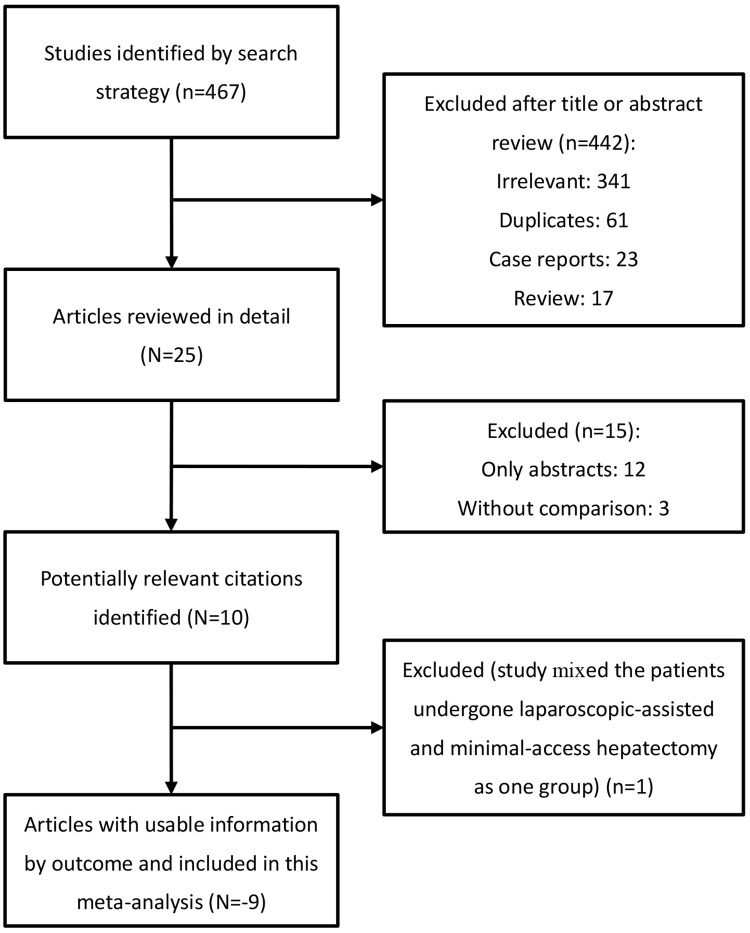
Selection flow diagram.

As shown in the flow chart (Fig 1), the search strategy initially generated 467 potentially relevant studies. After the titles and abstracts were retrieved, 442 articles were excluded. Twenty-five articles were reviewed in detail. Finally, 9 non-randomized comparative studies were included for the current meta-analysis[12, 13, 21–27].

### Study Characteristics

The characteristics of included studies are shown in Table 1. The nine studies included a total of 1346 patients: 318 in LLDH group and 1028 in OLDH group. Four studies were conducted in Korea[22, 23, 25, 27], one in the United States[21], one in France[12], one in China[24]., one in Japan[13], and one in India[26]. The sample size of the included studies ranged from 22 to 493 patients. Seven of the nine studies reported on the conversion rate in LLDH group, which varied from 0 to 10%[12, 13, 21–24, 26]. The reasons for conversion including a left portal branch injury (n = 1), a right hepatic vein injury (n = 1) and an inferior vena cava injury (n = 1). And the reasons for the other two conversions were not detailed described.

**Table 1:** Characteristics of studies included in the meta-analysis.

Study	Country	Study Design	Group	n	Male/female	Age (yr) (mean ± SD)	Matching^a^	Conversions (n [%])	Study quality
**Soubrane et al. (2006)**	France	Case control	LLDH	16	10/6	29 ± 5	1–5, 10, 12–14, 16	1 (6.25)	******
			OLDH	14	9/5	32 ± 5			
**Baker et al. (2009)**	United States	Case control	LLDH	33	15/18	37.0 ± 10.3	1–4, 6–9	2(6.06)	*******
			OLDH	33	13/20	39.1 ± 11.1			
**Kim et al. (2011)**	Korea	Case control	LLDH	11	1/10	29.6 ± 5.7	2, 4, 5, 10, 11, 13–15	0	******
			OLDH	11	6/5	35.2 ± 3.8			
**Choi. HJ et al. (2012)**	Korea	Case control	LLDH	20	12/8	29.7 ± 10.13	1–4, 10, 11, 14, 15, 17	2(10)	*******
			OLDH	90	58/32	36.8 ± 12.01			
**Marubashi et al. (2013)**	Japan	Prospective cohort	LLDH	31	13/18	35.8 ± 8.4	-	0	********
			OLDH	79	54/25	37.8 ± 10.1			
**Zhang et al. (2014)**	China	Prospective cohort	LLDH	25	13/12	37.2 ± 8.7	-	0	*******
			OLDH	25	14/11	37.4 ± 10.5			
**Choi. YR et al. (2014)**	Korea	Case control	LLDH	9	-	-	-	-	******
			OLDH	484	346/138	31.5			
**Makki et al. (2014)**	India	Case control	LLDH	26	13/13	27.46 ± 9.40	1 2 3 4	0	******
			OLDH	24	18/6	32.42 ± 8.47			
**Suh et al. (2015)**	Korea	Prospective cohort	LLDH	147	98/49	29.4 ± 8.5	-	-	*******
			OLDH	268	206/62	34.0 ± 9.7			

LLDH: laparoscopic living donor hepatectomy; OLDH: open living donor hepatectomy.

^a^1: age; 2: gender; 3: body mass index; 4: type of hepatectomy; 5: graft volume; 6: hepatic artery anomalies; 7: portal vein anomalies; 8: hepatic vein anomalies; 9: biliary anomalies; 10: ALT; 11: AST; 12: GGT; 13: total bilirubin; 14: hemoglobin; 15: prothrombin time; 16: prothrombin rate; 17: international normalized ratio.

### Meta-analysis Results

**Fig 2:**
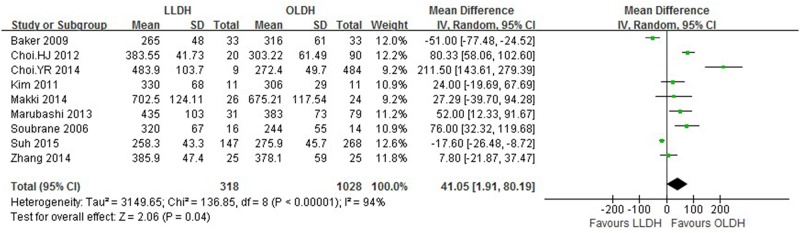
Forest plot displaying the results of the meta-analysis on operative time.

**Fig 3:**
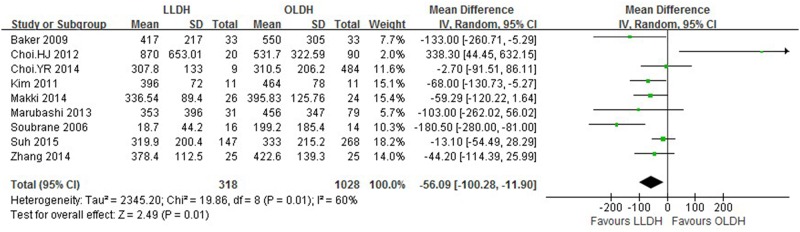
Forest plot displaying the results of the meta-analysis on intraoperative blood loss.

**Fig 4:**
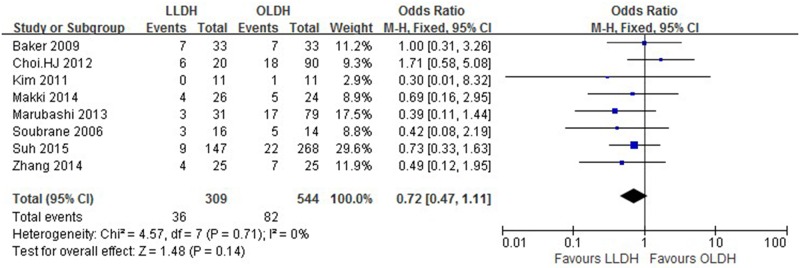
Forest plot displaying the results of the meta-analysis on overall complication.

**Fig 5:**
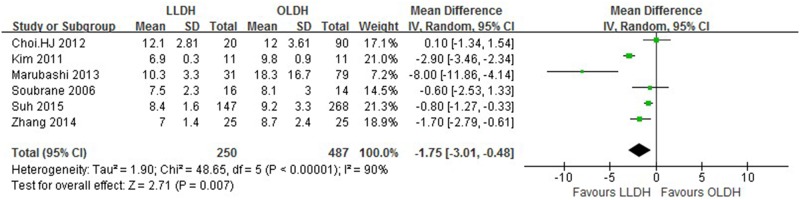
Forest plot displaying the results of the meta-analysis on hospital stay.

Results are presented in Figs 2–5 and summarized in Table 2.

**Table 2:** Results of meta-analysis comparing laparoscopic versus open living donor hepatectomy.

Outcome of interest	No. of studies	No. of donors	OR/WMD	95% CI	*P* value	*I*^2^ (%)
**Operative outcomes**						
Operation time (min)	9	1346	41.05	1.91, 80.19	0.04	94
Intraoperative blood loss (mL)	9	1346	-56.09	-100.28, -11.90	0.01	60
**Postoperative outcomes**						
Overall complication	8	853	0.72	0.47, 1.11	0.14	0
Bile leakage	4	256	0.59	0.15, 2.24	0.43	0
Postoperative bleeding	3	575	1.97	0.54, 7.22	0.31	0
Pulmonary complication	3	575	0.95	0.22, 4.04	0.94	35
Wound complication	5	627	0.55	0.20, 1.51	0.25	0
Hospital stay (day)	6	737	-1.75	-3.01, -0.48	0.007	90
Time to dietary intake	2	132	-0.03	-1.18, 1.12	0.96	94
Period of analgesic use	2	80	-0.52	-1.11, 0.06	0.08	48

OR: odds ratio, WMD: weighted mean difference; CI: confidence interval.

#### Operative Outcomes

Nine studies[12, 13, 21–27] provided mean operation time, analysis of which showed that LLDH had significantly longer operative time compared to OLDH (patients 1346; WMD: 41.05 min; 95%CI: 1.91–80.19 min; *P* = 0.04). Similarly, nine studies[12, 13, 21–27] reported detailed data for estimated blood loss between the two groups. We found that intraoperative blood loss was significantly less in LLDH group [patients 1346; WMD: -56.09 mL; 95%CI: -100.28-(-11.90) mL; *P* = 0.01]. There was significant heterogeneity of difference in operation time and blood loss between the studies (*P* < 0.1).

#### Postoperative Outcomes

Six studies[12, 13, 22–24, 27] reported on length of hospital stay, which was found to be significantly shorter in the LLDH group versus the OLDH group [patients 737; WMD: -1.75 d; 95%CI: -3.01-(-0.48) d; *P* = 0.007] with significant heterogeneity (*P* < 0.1). No significant difference was observed between the groups regarding other outcomes, such as overall complication (trials 8; patients 853; OR: 0.72; 95%CI: 0.47–1.11; *P* = 0.14), bile leakage (trials 4; patients 256; OR: 0.59; 95%CI: 0.15–2.24; *P* = 0.43), postoperative bleeding (trials 3; patients 575; OR: 1.97; 95%CI: 0.54–7.22; *P* = 0.31), pulmonary complication (trials 3; patients 575; OR: 0.95; 95%CI: 0.22–4.04; *P* = 0.94), wound complication (trials 5; patients 627; OR: 0.55; 95%CI: 0.20–1.51; *P* = 0.25), time to dietary intake (trials 2; patients 132; WMD: -0.03; 95%CI: -1.18–1.12; *P* = 0.96) and period of analgesic use (trials 2; patients 80; WMD: -0.52; 95%CI: -1.11–0.06; *P* = 0.08).

### Publication Bias

**Fig 6:**
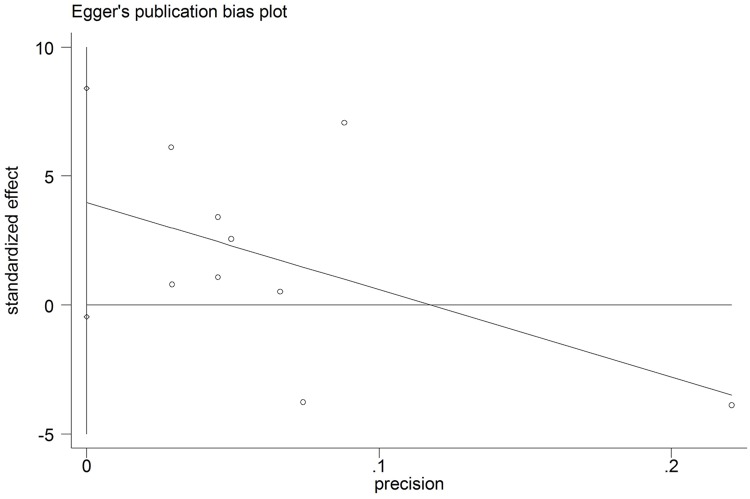
Egger test results of studies on operation time.

**Fig 7:**
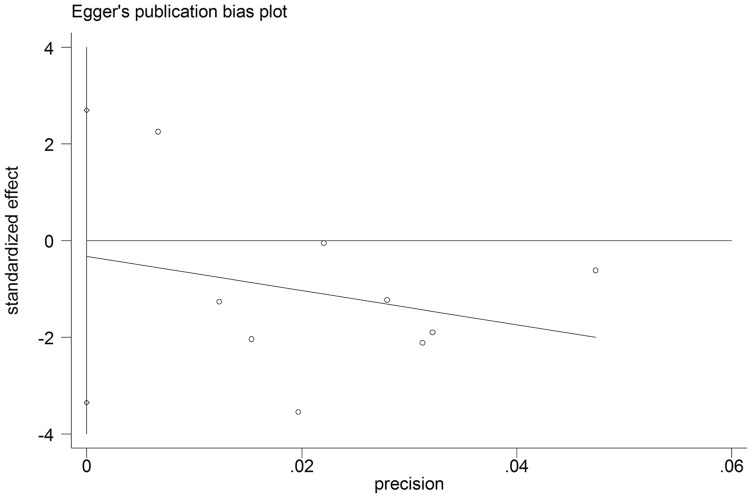
Egger test results of studies on intraoperative blood loss.

**Fig 8:**
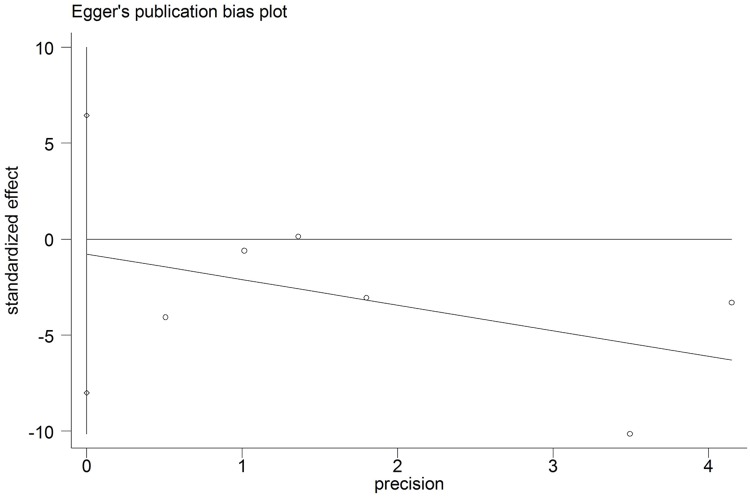
Egger test results of studies on hospital stay.

The funnel plot was based on the operation time, intraoperative blood loss and hospital stay, and the appearance was symmetrical. The Egger test gave a *P* value of 0.072 for operation time, a *P* value of 0.804 for intraoperative blood loss and a *P* value of 0.781 for hospital stay, indicating no evidence of publication bias (Figs 6–8).

## Discussion

Although OLDH is still the standard procedure for LDLT, LLDH has been designed to perform a less invasive donor operation[28]. LLDH is a challenging technique for surgeons because the liver is an unpaired organ with the need for parenchymal transection[11]. Rare but catastrophic complications have been reported in LLDH[29, 30]. On the contrary, increased experience in donor surgery and technical advancements have reduced the incidence of donor morbidity and mortality[29, 31]. In spite of these advancements, LLDH has not been accepted by many surgeons, mainly for reasons of unassured safety[15]. However, the difference in perioperative outcomes between LLDH and OLDH has not been assessed in a randomized controlled trial.

Our results demonstrated that LLDH was associated with significantly longer operative time, which could be explained by the frequent installation and removal of laparoscopic devices, mobilization and dissection the lobe of the liver under laparoscopy as well as surgeons’ experience and initial learning curve[26, 27]. Meanwhile, the operative time difference between LLDH and OLDH might correlate with the type of hepatectomy. Marubashi et al. reported that operative time in the left-lobe laparoscopy-assisted hybrid donor hepatectomy was associated with the maximal distance between the surface of the right lobe and the portal vein bifurcation[13]. Decreasing the operating time was not a crucial aim in LLDH, but operating time was indeed both related to the warm ischemia time and some rare complications such as gas embolism[32]. The meta-analysis also revealed decreased blood loss for LLDH compared to OLDH. This finding was probably attributed to the more meticulous dissection under image magnification and smaller incision on the abdominal wall provided by the laparoscopic approach as well as the hemostatic effect of pneumoperitoneum on the hepatic vein branches[33].

The efficacy of the LDLT should prioritize the donor safety[15]. A recent worldwide survey revealed that the donor mortality rate was 0.20% (23/11553), with 19 of these 23 deaths related to the surgical procedure[30]. In this analysis, we found no mortality in neither of the donor groups. However, the LLDH group reported five cases that conversed to open surgery, three of which the causative data were available. One case was converted to open donation in left lateral sectionectomy when a left portal branch injury occurred[12], and other two cases of conversion due to a right hepatic vein injury and an inferior vena cava injury in right hepatectomy[23]. Additionally, we found no significant difference in postoperative morbidity between the two groups with regards to developing overall complication, bile leakage, bleeding, pulmonary complication and wound complication. These results pointed towards the feasibility of LLDH.

With respect to postoperative recovery, the length of hospital stay after surgery was shorter in the LLDH group. This might be ascribed to the surgeons’ postoperative protocols. However, our results revealed no significant difference between LLDH and OLDH regarding the period of analgesic use and time to dietary intake. Thus, combined with the previous results about the postoperative complications, the laparoscopic method of incision seemed to be in favor of reducing hospital stay.

Although our analysis overcomes the drawbacks of each individual study and may provide the most convincing results so far, it has some potential limitations. First, significant heterogeneity was found in certain outcome measures, which might have resulted from differences in study designs, sample sizes, geographical variations, donors’ baseline characteristics, surgical techniques and surgeons’ learning curve. For example, there was no randomized trials on our topic, all of them were observational. Also, both cohort studies and case-control studies were included in this meta-analysis. The six case-control studies involved in our meta-analysis were all hospital based[12, 21–23, 26, 27]. Meanwhile, several studies with small sample size brought some concerns regarding the reliability of their results[12, 13, 21–26]. To address this issue, we applied the random-effects model to determine the overall estimate of variability.

Second, we did a mixed analysis and did not differentiate laparoscopy-assisted hybrid donor hepatectomy or totally laparoscopic donor hepatectomy, as well as left hepatectomy or right hepatectomy. Because most studies had mixed results only and the data in each group was insufficient for analysis.

Third, it should be noted that donors who underwent LLDH were a selected population. For example, LLDH should only be performed in cases with a favorable anatomy[10], whereas OLDH was preferred for urgent LDLT from some surgeon’s view point.

Fourth, the outcome of the operation time had some sensitivity. In view of the mainly influencing factors, we tried subgroup analyses classified by year or type of hepatectomy. However, it was unable to adequately explore sources of this heterogeneity, and meta-regression was not possible for the small number of studies. Therefore, the meta-analysis results of operation time should be carefully concluded. Fortunately, it did not affect the pooled results of intraoperative blood loss and hospital stay.

Finally, although the Egger test showed no evidence of publication bias on the main outcome measures, for comparisons of the time to dietary intake and period of analgesic use, the analysis was based on only 2 studies. Under these circumstances, a single study might have great impact on the pooled results, so the potential bias due to publication could not be minimized. Based on these limitations, more future prospective studies should be needed.

In conclusion, our meta-analysis suggests that LLDH is a safe and effective alternative to OLDH for donors. The promising use of LLDH could further minimize its invasiveness and benefit donors’ postoperative recovery. Further prospective randomized controlled studies may add more information to ascertain the advantages of LLDH in LDLT.

## Supporting Information

S1 Checklist PRISMA Checklist. (DOC)

S1 Fig Forest plot results not shown in the manuscript. (PDF)

S1 File PRISMA Flow Diagram. (DOC)

S2 File The 341 irrelevant articles in Selection flow diagram. (RTF)

S3 File The results of sensitivity analyses. (RAR)
