# Supplementary material for: Meta-Analysis of Laparoscopic versus Open Hepatectomy for Live Liver Donors
Source: PLoS One. 2016 Oct 27;11(10):e0165319. doi: 10.1371/journal.pone.0165319 (PMC5082914; doi:10.1371/journal.pone.0165319)
Supplement: S1 Fig — (PDF) [file pone.0165319.s002.pdf]

The other forest plot results not shown in the manuscript:

Study or Subgroup	LLDH Events	LLDH Total	OLDH Events	OLDH Total	Weight	Odds Ratio M-H, Fixed, 95% CI
Baker 2009	1	33	1	33	16.1%	1.00 [0.06, 16.69]
Choi.HJ 2012	0	20	8	90	51.5%	0.24 [0.01, 4.27]
Soubrane 2006	1	16	0	14	8.0%	2.81 [0.11, 74.56]
Zhang 2014	0	25	1	25	24.4%	0.32 [0.01, 8.25]
**Total (95% CI)**		**94**		**162**	**100.0%**	**0.59 [0.15, 2.24]**
Total events	2		10			

Heterogeneity: Chi² = 1.52, df = 3 (P = 0.68); I² = 0%
Test for overall effect: Z = 0.78 (P = 0.43)

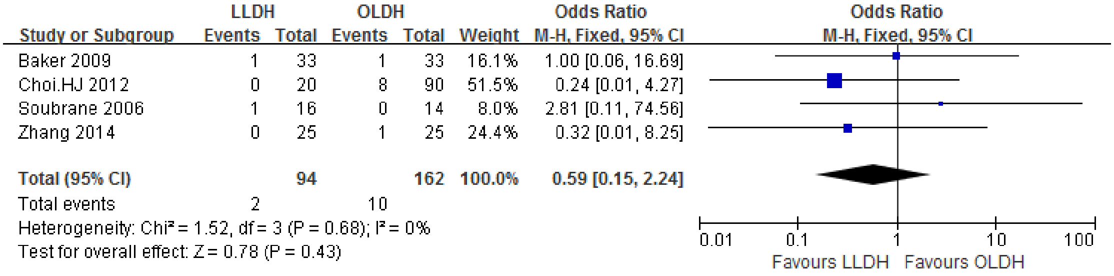

Figure a Forest plot displaying the results of the meta-analysis on bile leakage.

Study or Subgroup	LLDH Events	LLDH Total	OLDH Events	OLDH Total	Weight	Odds Ratio M-H, Fixed, 95% CI
Choi.HJ 2012	0	20	1	90	17.7%	1.46 [0.06, 37.02]
Suh 2015	3	147	3	268	67.1%	1.84 [0.37, 9.24]
Zhang 2014	1	25	0	25	15.2%	3.12 [0.12, 80.39]
**Total (95% CI)**		**192**		**383**	**100.0%**	**1.97 [0.54, 7.22]**
Total events	4		4			

Heterogeneity: Chi² = 0.12, df = 2 (P = 0.94); I² = 0%
Test for overall effect: Z = 1.02 (P = 0.31)

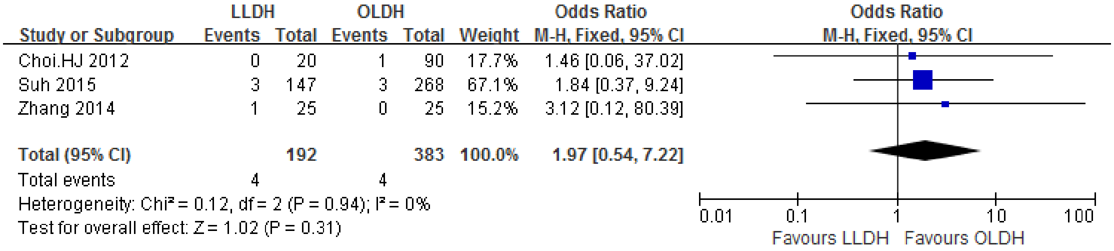

Figure b Forest plot displaying the results of the meta-analysis on postoperative bleeding.

Study or Subgroup	LLDH Events	LLDH Total	OLDH Events	OLDH Total	Weight	Odds Ratio M-H, Random, 95% CI
Choi.HJ 2012	2	20	4	90	39.1%	2.39 [0.41, 14.05]
Suh 2015	0	147	6	268	20.0%	0.14 [0.01, 2.45]
Zhang 2014	3	25	3	25	40.9%	1.00 [0.18, 5.51]
**Total (95% CI)**		**192**		**383**	**100.0%**	**0.95 [0.22, 4.04]**
Total events	5		13			

Heterogeneity: Tau² = 0.59; Chi² = 3.09, df = 2 (P = 0.21); I² = 35%
Test for overall effect: Z = 0.08 (P = 0.94)

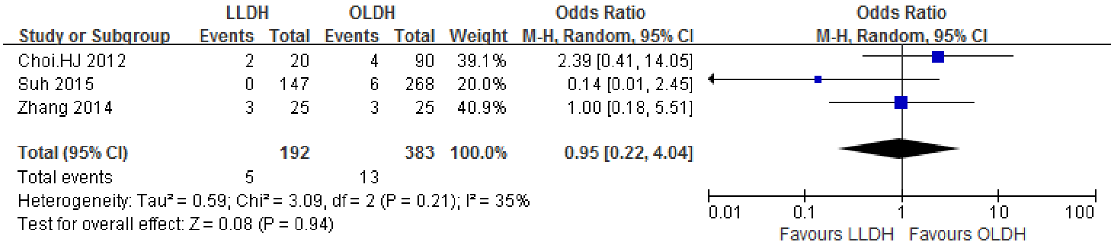

Figure c Forest plot displaying the results of the meta-analysis on pulmonary complication

Study or Subgroup	LLDH Events	LLDH Total	OLDH Events	OLDH Total	Weight	Odds Ratio M-H, Fixed, 95% CI
Choi.HJ 2012	2	20	5	90	14.5%	1.89 [0.34, 10.52]
Kim 2011	0	11	1	11	12.7%	0.30 [0.01, 8.32]
Soubrane 2006	2	16	2	14	16.5%	0.86 [0.10, 7.04]
Suh 2015	0	147	5	268	34.5%	0.16 [0.01, 2.96]
Zhang 2014	0	25	2	25	21.7%	0.18 [0.01, 4.04]
**Total (95% CI)**		**219**		**408**	**100.0%**	**0.55 [0.20, 1.51]**
Total events	4		15			

Heterogeneity: Chi² = 3.44, df = 4 (P = 0.49); I² = 0%
Test for overall effect: Z = 1.16 (P = 0.25)

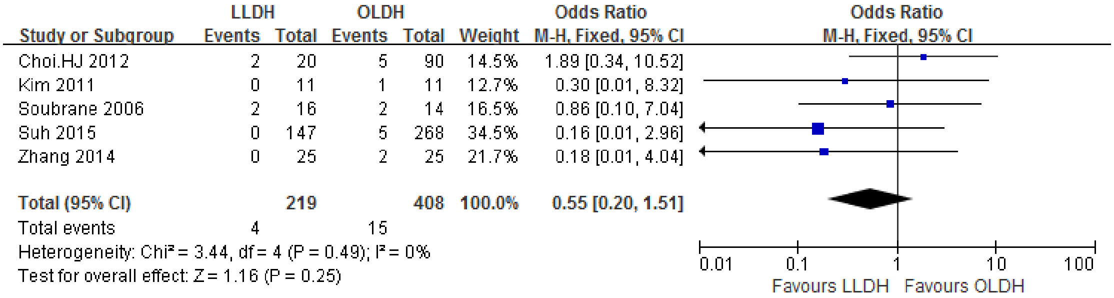

Figure d Forest plot displaying the results of the meta-analysis on wound complication

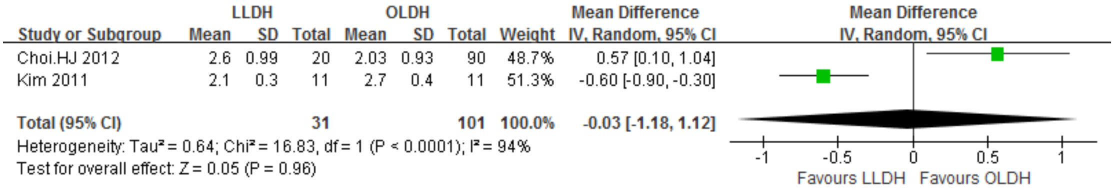

Figure e Forest plot displaying the results of the meta-analysis on time to dietary intake.

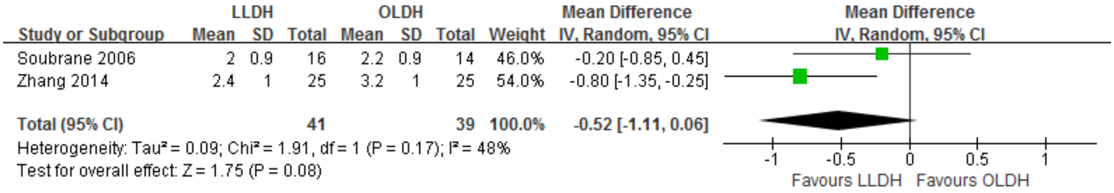

Figure f Forest plot displaying the results of the meta-analysis on period of analgesic use.
