# Supplementary material for: Meta-Analysis of Laparoscopic versus Open Hepatectomy for Live Liver Donors
Source: PLoS One. 2016 Oct 27;11(10):e0165319. doi: 10.1371/journal.pone.0165319 (PMC5082914; doi:10.1371/journal.pone.0165319)
Supplement: S1 File — (DOC) [file pone.0165319.s003.doc]

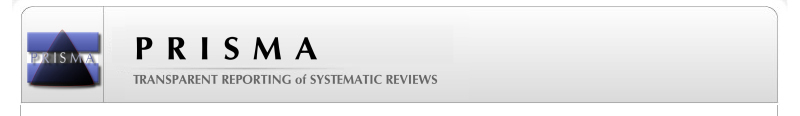
**PRISMA 2009 Flow Diagram**

**Screening**

**Included**

**Eligibility**

**Identification**

Records identified through database searching
(n =467)

Additional records identified through other sources
(n = 0)

Records after duplicates removed
(n =406)

Records screened
(n =406)

Records excluded
(n =381)

Full-text articles assessed for eligibility
(n =25)

Full-text articles excluded, with reasons
(n =15)

Studies included in qualitative synthesis
(n = 10)

Studies included in quantitative synthesis (meta-analysis)
(n = 9)
