# Supplementary material for: Meta-Analysis of Laparoscopic versus Open Hepatectomy for Live Liver Donors
Source: PLoS One. 2016 Oct 27;11(10):e0165319. doi: 10.1371/journal.pone.0165319 (PMC5082914; doi:10.1371/journal.pone.0165319)
Supplement: S2 File — (RTF) [file pone.0165319.s004.rtf]

	1. Laparoscopic liver resection (Structured abstract) Health Technology Assessment Database, 2005:2.
	2. Swiss Society Of Radiology and the Swiss Society of Nuclear Medicine 2014, SGR 2014. Nuklearmedizin 53, A111 (2014).
	3. Acosta F. et al. Right ventricular function during the anhepatic phase in liver transplantation Transplantation proceedings, vol. 26, 1994:3671-3672.
	4. Afaneh C., Kluger M. D. Laparoscopic liver resection: Lessons at the end of the second decade. Seminars in Liver Disease 33, 226-235 (2013).
	5. Aikot S., Goyal N., Wadhavan M., Gupta S. Previous cholecystectomy is not a contraindication for right lobe liver donation. Liver Transplantation 19, S209 (2013).
	6. Akhtar M. M., Orakzai N., Qureshi A. M. Transurethral electric vaporisation of prostate as an alternate to trans urethral resection in benign prostatic hyperplasia (Structured abstract) Journal of Ayub Medical College, vol. 16, 2004:16-20.
	7. Akl Elie A. et al. Anticoagulation for people with cancer and central venous catheters Cochrane Database of Systematic Reviews: John Wiley & Sons, Ltd, 2014.
	8. Akyol M. Comparison of open and laparoscopic live donor left lateral sectionectomy (Br J Surg 2011; 98: 1302-1308). British Journal of Surgery 98, 1308 (2011).
	9. Alhasso A., Glazener Cathryn M. A., Pickard R., N'Dow James M. O. Adrenergic drugs for urinary incontinence in adults Cochrane Database of Systematic Reviews: John Wiley & Sons, Ltd, 2005.
	10. Allen Victoria B., Gurusamy Kurinchi S., Takwoingi Y., Kalia A., Davidson Brian R. Diagnostic accuracy of laparoscopy following computed tomography (CT) scanning for assessing the resectability with curative intent in pancreatic and periampullary cancer Cochrane Database of Systematic Reviews: John Wiley & Sons, Ltd, 2013.
	11. Als-Nielsen B., Koretz Ronald L., Gluud Lise L., Gluud C. Branched-chain amino acids for hepatic encephalopathy Cochrane Database of Systematic Reviews: John Wiley & Sons, Ltd, 2003.
	12. Ang C., Chan Karen K. L., Bryant A., Naik R., Dickinson Heather O. Ultra-radical (extensive) surgery versus standard surgery for the primary cytoreduction of advanced epithelial ovarian cancer Cochrane Database of Systematic Reviews: John Wiley & Sons, Ltd, 2011.
	13. Arezzo A., Passera R., Scozzari G., Verra M., Morino M. Laparoscopy for extraperitoneal rectal cancer reduces short-term morbidity. Results of a systematic reviewand meta-analysis Digestive and liver disease, vol. 45, 2013:S180.
	14. Awad T., Thorlund K., Gluud C. Cryotherapy for hepatocellular carcinoma Cochrane Database of Systematic Reviews: John Wiley & Sons, Ltd, 2009.
	15. Bala Malgorzata M., Riemsma Robert P., Wolff R., Kleijnen J. Cryotherapy for liver metastases Cochrane Database of Systematic Reviews: John Wiley & Sons, Ltd, 2013.
	16. Bala Malgorzata M., Riemsma Robert P., Wolff R., Kleijnen J. Microwave coagulation for liver metastases Cochrane Database of Systematic Reviews: John Wiley & Sons, Ltd, 2013.
	17. Barbaros U. et al. Evaluation of clinical results of single-incision laparoscopic surgeries in general surgery Surgical Endoscopy and Other Interventional Techniques, vol. 28, 2014:S80.
	18. Barone Damiano G., Lawrie Theresa A., Hart Michael G. Image guided surgery for the resection of brain tumours Cochrane Database of Systematic Reviews: John Wiley & Sons, Ltd, 2014.
	19. Bartlett A., Rela M. Progress in surgical techniques in pediatric liver transplantation. Pediatric Transplantation 14, 33-40 (2010).
	20. Bath Philip M. W., Krishnan K. Interventions for deliberately altering blood pressure in acute stroke Cochrane Database of Systematic Reviews: John Wiley & Sons, Ltd, 2014.
	21. Belghiti J., Durand F. Hepatectomy vs. liver transplantation: A combination rather than a opposition. Liver Transplantation 13, 636-638 (2007).
	22. Belluomini M. A. et al. The robotic right hepatectomy. HPB 16, 702-703 (2014).
	23. Berber E. et al. Initial experience with a new articulating energy device for laparoscopic liver resection Surgical Endoscopy and Other Interventional Techniques, vol. 28, 2014:974-978.
	24. Berdejo L. Transhiatal versus transthoracic esophagectomy for clinical stage I esophageal carcinoma Hepato-gastroenterology, vol. 42, 1995:789-791.
	25. Bergamaschi R., Arnaud J. P. Immediately recognizable benefits and drawbacks after laparoscopic colon resection for benign disease (Structured abstract) Surgical Endoscopy - Ultrasound and Interventional Techniques, vol. 11, 1997:802-804.
	26. Bhojani F. D. et al. Clinical and economic comparison of laparoscopic to open liver resections using a 2-to-1 matched pair analysis: an institutional experience (Provisional abstract) Journal of the American College of Surgeons, vol. 214, 2012:184-195.
	27. Bismuth H., Majno P. E. Hepatobiliary surgery. Journal of Hepatology, Supplement 32, 208-224 (2000).
	28. Bjelakovic G. et al. Vitamin D supplementation for prevention of mortality in adults Cochrane Database of Systematic Reviews: John Wiley & Sons, Ltd, 2014.
	29. Bjelakovic G., Nikolova D., Gluud Lise L., Simonetti Rosa G., Gluud C. Antioxidant supplements for prevention of mortality in healthy participants and patients with various diseases Cochrane Database of Systematic Reviews: John Wiley & Sons, Ltd, 2012.
	30. Bohm B., Junghans T., Neudecker J., Schwenk W. Hepatic and renal function following laparoscopic and conventionell resection of colorectal cancer - Results from a prospective randomized trial. [German] Viszeralchirurgie, vol. 34, 1999:20-24.
	31. Borle D. P., Bharathy K. G. S., Kumar S., Pamecha V. Laparoscopic living donor left hepatectomy: Donor safety remains the overriding concern. American Journal of Transplantation 14, 735 (2014).
	32. Boucaud C. et al. Peripheral deep venous thromboses in living liver donors: Efficiency of a simple non invasive procedure. Liver Transplantation 16, S216 (2010).
	33. Boyvat F. Biliary interventions for benign diseases. CardioVascular and Interventional Radiology 33, 118-119 (2010).
	34. Brito Poveda V., Martinez E. Z., Galvao C. M. Active cutaneous warming systems to prevent intraoperative hypothermia: a systematic review (Structured abstract) Revista Latino-Americana de Enfermagem, vol. 20, 2012:183-191.
	35. Buell J. F. et al. The international position on laparoscopic liver surgery: The Louisville Statement, 2008. Ann Surg 250, 825-830 (2009).
	36. Buell J. F. et al. Experience with more than 500 minimally invasive hepatic procedures (Provisional abstract) Annals of Surgery, vol. 248, 2008:475-486.
	37. Cammu G. et al. Anaesthetic management and outcome in right-lobe living liver-donor surgery. European Journal of Anaesthesiology 19, 93-98 (2002).
	38. Caniglia F. et al. Laparoscopic robot-assisted major hepatectomy. HPB 14, 561 (2012).
	39. Cannon R. M. et al. Financial comparison of laparoscopic versus open hepatic resection using deviation-based cost modeling (Provisional abstract) Annals of Surgical Oncology, vol. 20, 2013:2887-2892.
	40. Carrafiello G. et al. How to select patients for tumour ablation Cardiovascular and interventional radiology, vol. 35, 2012:S95-s97.
	41. Catalano O. A. et al. Vascular and biliary variants in the liver: implications for liver surgery. Radiographics : a review publication of the Radiological Society of North America, Inc 28, 359-378 (2008).
	42. Cauchy F., Schwarz L., Scatton O., Soubrane O. Laparoscopic liver resection for living donation: where do we stand? World Journal of Gastroenterology 20, 15590-15598 (2014).
	43. Cha C. H., Saif M. W., Yamane B. H., Weber S. M. Hepatocellular Carcinoma: Current Management. Current Problems in Surgery 47, 10-67 (2010).
	44. Chapman W. C. Liver paper summary: IHPBA meeting 2004. Washington, DC. HPB 7, 164 (2005).
	45. Charlton S., Cyna Allan M., Middleton P., Griffiths James D. Perioperative transversus abdominis plane (TAP) blocks for analgesia after abdominal surgery Cochrane Database of Systematic Reviews: John Wiley & Sons, Ltd, 2010.
	46. Chavez-Tapia Norberto C. et al. Bariatric surgery for non-alcoholic steatohepatitis in obese patients Cochrane Database of Systematic Reviews: John Wiley & Sons, Ltd, 2010.
	47. Chen K. H., Wu J. M., Cheng K. H. Laparoscopic right hepatectomy: Selective hilum dissection technique. Surgical Endoscopy and Other Interventional Techniques 27, S410 (2013).
	48. Cheng X. et al. Transarterial (chemo)embolization for curative resection of hepatocellular carcinoma: a systematic review and meta-analyses (Provisional abstract) Database of Abstracts of Reviews of Effects, 2014:1159-1170.
	49. Cherian P. T. et al. Laparoscopic liver resection: Wedge resections to living donor hepatectomy, are we heading in the right direction? World Journal of Gastroenterology 20, 13369-13381 (2014).
	50. Cherqui D., Belghiti J. Hepatic surgery. What progress? What future? Gastroenterologie Clinique et Biologique 33, 896-902 (2009).
	51. Chowdhury A., Varadhan K., Neal K., Lobo D. Perioperative prebiotics, probiotics or synbiotics for elective abdominal surgery in adults Cochrane Database of Systematic Reviews: John Wiley & Sons, Ltd, 2011.
	52. Cipolletta L. et al. A randomised study of hydro-jet vs. needle injection for lifting colorectal lesions prior to endoscopic resection Digestive and liver disease : official journal of the Italian Society of Gastroenterology and the Italian Association for the Study of the Liver, vol. 42, 2010:127-130.
	53. Cirocchi R. et al. Non-resection versus resection for an asymptomatic primary tumour in patients with unresectable Stage IV colorectal cancer Cochrane Database of Systematic Reviews: John Wiley & Sons, Ltd, 2012.
	54. Cirocchi R. et al. Radiofrequency ablation in the treatment of liver metastases from colorectal cancer Cochrane Database of Systematic Reviews: John Wiley & Sons, Ltd, 2012.
	55. Ciuni R. et al. Nutritional aspects in patient undergoing liver resection. Updates in surgery 63, 249-252 (2011).
	56. Colquitt Jill L., Pickett K., Loveman E., Frampton Geoff K. Surgery for weight loss in adults Cochrane Database of Systematic Reviews: John Wiley & Sons, Ltd, 2014.
	57. Coolsen M. Enhanced recovery after surgery (ERAS) in HPB disease Hpb, vol. 16, 2014:46-47.
	58. Costi R. et al. Lessons learnt from the first 100 laparoscopic liver resections: Not delaying conversion may allow reducing blood loss and operative time. Surgical Endoscopy and Other Interventional Techniques 26, S134 (2012).
	59. Croome K. P., Yamashita M. H. Laparoscopic vs open hepatic resection for benign and malignant tumors: an updated meta-analysis (Structured abstract) Archives of Surgery, vol. 145, 2010:1109-1118.
	60. Crovari F. et al. Ovine model for advanced simulation training in laparoscopic liver surgery. HPB 14, 574 (2012).
	61. Cuomo O. et al. Living Donor Liver Transplantation: Early Single-Center Experience. Transplantation Proceedings 38, 1101-1105 (2006).
	62. Cuschieri A. Laparoscopic surgery in Europe. Where are we going? Cirugía española 79, 10-21 (2006).
	63. Dam R. M. et al. Open versus laparoscopic left lateral hepatic sectionectomy within an enhanced recovery ERAS programme (ORANGE II - Trial): Study protocol for a randomised controlled trial Trials, vol. 13, 2012.
	64. Dat Anthony D., Poon F. Robotic surgery for rectal cancer Cochrane Database of Systematic Reviews: John Wiley & Sons, Ltd, 2011.
	65. De Caluwé L., Van Nieuwenhove Y., Ceelen Wim P. Preoperative chemoradiation versus radiation alone for stage II and III resectable rectal cancer Cochrane Database of Systematic Reviews: John Wiley & Sons, Ltd, 2013.
	66. Deschamps F. Combining transcatheter and systemic chemotherapy Cardiovascular and interventional radiology, vol. 35, 2012:S79-s81.
	67. Detry O. et al. Recent advances in liver surgery and transplantation. Revue Medicale de Liege 62, 310-316 (2007).
	68. Deva S., Jameson M. Histamine type 2 receptor antagonists as adjuvant treatment for resected colorectal cancer Cochrane Database of Systematic Reviews: John Wiley & Sons, Ltd, 2012.
	69. Dinis-Ribeiro M., Costa-Pereira A., Lopes C., Moreira-Dias L. Feasibility and cost-effectiveness of using magnification chromoendoscopy and pepsinogen serum levels for the follow-up of patients with atrophic chronic gastritis and intestinal metaplasia (Structured abstract) Journal of Gastroenterology and Hepatology, vol. 22, 2007:1594-1604.
	70. Dong Z., Xu J., Wang Z., Petrov Maxim S. Stents for the prevention of pancreatic fistula following pancreaticoduodenectomy Cochrane Database of Systematic Reviews: John Wiley & Sons, Ltd, 2013.
	71. Douard R. et al. A two-step strategy for enlargement of left arterial branch in a living related liver graft with dual arterial supply. Transplantation 73, 993-994 (2002).
	72. Duan J., Yue H., Liu K., Wu M., Yang J. Percutaneous radiofrequency ablation versus repeat hepatectomy for recurrent hepatocellular carcinoma: retrospective randomized control study (Provisional abstract) Journal of Medical Colleges of PLA, vol. 26, 2011:316-323.
	73. Eguchi S. et al. Elective living donor liver transplantation by hybrid hand-assisted laparoscopic surgery and short upper midline laparotomy. Surgery 150, 1002-1005 (2011).
	74. Eke Ahizechukwu C., Chawla M., Bridges N., Ezebialu I. Progestogen only versus combined oral contraceptive pills for fibroid related heavy menstrual bleeding Cochrane Database of Systematic Reviews: John Wiley & Sons, Ltd, 2012.
	75. Elattar A., Bryant A., Winter-Roach Brett A., Hatem M., Naik R. Optimal primary surgical treatment for advanced epithelial ovarian cancer Cochrane Database of Systematic Reviews: John Wiley & Sons, Ltd, 2011.
	76. Elwood D., Pomposelli J. J. Hepatobiliary Surgery: Lessons Learned from Live Donor Hepatectomy. Surgical Clinics of North America 86, 1207-1217 (2006).
	77. Erickson R. A., Garza A. A. EUS with EUS-guided fine-needle aspiration as the first endoscopic test for the evaluation of obstructive jaundice (Structured abstract) Gastrointestinal Endoscopy, vol. 53, 2001:475-484.
	78. Esposito M. et al. Interventions for replacing missing teeth: horizontal and vertical bone augmentation techniques for dental implant treatment Cochrane Database of Systematic Reviews: John Wiley & Sons, Ltd, 2009.
	79. Fan N., Yang G. S., Lu J. H., Yang N., Zhang H. B. Oral administration of geranylgeranylacetone plus local somatothermal stimulation: A simple, effective, safe and operable preconditioning combination for conferring tolerance against ischemia-reperfusion injury in rat livers. World Journal of Gastroenterology 11, 5725-5731 (2005).
	80. Fancellu A. et al. Meta-analysis of trials comparing minimally-invasive and open liver resections for hepatocellular carcinoma (Provisional abstract) Journal of Surgical Research, vol. 171, 2011:e33-e45.
	81. Farquhar C., Marjoribanks J., Basser R., Lethaby A. High dose chemotherapy and autologous bone marrow or stem cell transplantation versus conventional chemotherapy for women with early poor prognosis breast cancer Cochrane Database of Systematic Reviews: John Wiley & Sons, Ltd, 2005.
	82. Fayek S., Aranovish D., Barth R., Philosophe B. Minimizing port sites for total laparoscopic left lateral hepatic segmentectomy using nulllessnull approach. HPB 14, 553 (2012).
	83. Fedorowicz Z., Lodge M., Al-asfoor A., Carter B. Resection versus no intervention or other surgical interventions for colorectal cancer liver metastases Cochrane Database of Systematic Reviews: John Wiley & Sons, Ltd, 2008.
	84. Feng K. et al. A randomized controlled trial of radiofrequency ablation and surgical resection in the treatment of small hepatocellular carcinoma Journal of hepatology, vol. 57, 2012:794-802.
	85. Feroci F. et al. Laparoscopic surgery improves postoperative outcomes in high-risk patients with colorectal cancer Surgical Endoscopy and Other Interventional Techniques, vol. 27, 2013:1130-1137.
	86. Figueras J. et al. Complete versus selective portal triad clamping for minor liver resections: a prospective randomized trial Annals of surgery, vol. 241, 2005:582-590.
	87. Figueredo A., Coombes Megan E., Mukherjee S. Adjuvant Therapy for completely resected Stage II Colon Cancer Cochrane Database of Systematic Reviews: John Wiley & Sons, Ltd, 2008.
	88. Findlay M. et al. A pilot study of preoperative and postoperative chemotherapy in patients with operable gastric cancer: Australasian Gastrointestinal Trials Group Study 9601 ANZ journal of surgery, vol. 77, 2007:247-252.
	89. Fioole B. et al. Additional value of contrast enhanced intraoperative ultrasound for colorectal liver metastases European journal of radiology, vol. 67, 2008:169-176.
	90. Fix O. K. et al. Five-year intention-to-treat outcome of down-staging of hepatocellular carcinoma prior to liver transplantation. Hepatology 50, 305A (2009).
	91. Fretland A. A. et al. The oslo comet-study: Randomized controlled study of open and laparoscopic liver resection for colorectal liver metastases Surgical Endoscopy and Other Interventional Techniques, vol. 28, 2014:S9.
	92. Fretland A. A. et al. Open liver resection for colorectal metastases induced a significant increase in interleukin-6 as compared to laparoscopic technique Hpb, vol. 16, 2014:250-251.
	93. Furness S. et al. Interventions for the treatment of oral cavity and oropharyngeal cancer: chemotherapy Cochrane Database of Systematic Reviews: John Wiley & Sons, Ltd, 2011.
	94. Galindo J. et al. Totally laparoscopic anatomical liver resections. Surgical results. Surgical Endoscopy and Other Interventional Techniques 27, S407 (2013).
	95. Ge L. et al. [Evaluation of short-term efficacy and safety after laparoscopic resection for mid-low rectal cancer] Zhonghua yi xue za zhi, vol. 92, 2012:98-101.
	96. George R. et al. Interventions for the treatment of metastatic extradural spinal cord compression in adults Cochrane Database of Systematic Reviews: John Wiley & Sons, Ltd, 2008.
	97. Gillams A. Radiofrequency ablation Cardiovascular and interventional radiology, vol. 36, 2013:S175.
	98. Gillams A. Will radiofrequency ablation replace surgery for small liver metastases? Cardiovascular and interventional radiology, vol. 37, 2014:S157-s159.
	99. Giulianotti P. C. et al. Robot-assisted right lobe donor hepatectomy. Transpl Int 25, e5-9 (2012).
	100. Gomez D. et al. Impact of specialized multi-disciplinary approach and an integrated pathway on outcomes in hilar cholangiocarcinoma European journal of surgical oncology, vol. 40, 2014:77-84.
	101. Govindaraju P., Goyal N., Wadhavan M., Gupta S. Post cholecystectomy status is not a contraindication for right lobe liver donation. Liver Transplantation 20, S324-S325 (2014).
	102. Grewal H. P. Impact of surgical innovation on liver transplantation. Lancet 359, 368-370 (2002).
	103. Grocott Michael P. W. et al. Perioperative increase in global blood flow to explicit defined goals and outcomes following surgery Cochrane Database of Systematic Reviews: John Wiley & Sons, Ltd, 2012.
	104. Grossmann E. M., Johnson F. E., Virgo K. S., Longo W. E., Fossati R. Follow-up of colorectal cancer patients after resection with curative intent-the GILDA trial Surgical oncology, vol. 13, 2004:119-124.
	105. Gruttadauria S. et al. Evolution of surgical approach to the venous system in conventional open right hepatectomy for living liver donation over a 10 years period in a single center. Liver Transplantation 19, S151-S152 (2013).
	106. Güenaga Katia F., Matos D., Wille-Jørgensen P. Mechanical bowel preparation for elective colorectal surgery Cochrane Database of Systematic Reviews: John Wiley & Sons, Ltd, 2011.
	107. Gurumurthy M., Bryant A., Shanbhag S. Effectiveness of different treatment modalities for the management of adult-onset granulosa cell tumours of the ovary (primary and recurrent) Cochrane Database of Systematic Reviews: John Wiley & Sons, Ltd, 2014.
	108. Gurusamy Kurinchi S. Management strategies for pancreatic pseudocysts: a network meta-analysis Cochrane Database of Systematic Reviews: John Wiley & Sons, Ltd, 2014.
	109. Gurusamy Kurinchi S. Enhanced recovery protocols for major upper gastrointestinal, liver and pancreatic surgery Cochrane Database of Systematic Reviews: John Wiley & Sons, Ltd, 2014.
	110. Gurusamy Kurinchi S., Davidson Brian R. Imaging modalities for characterising focal pancreatic lesions Cochrane Database of Systematic Reviews: John Wiley & Sons, Ltd, 2012.
	111. Gurusamy Kurinchi S., Kumar S., Davidson Brian R. Prophylactic gastrojejunostomy for unresectable periampullary carcinoma Cochrane Database of Systematic Reviews: John Wiley & Sons, Ltd, 2013.
	112. Gurusamy Kurinchi S., Kumar Y., Pamecha V., Sharma D., Davidson Brian R. Ischaemic pre-conditioning for elective liver resections performed under vascular occlusion Cochrane Database of Systematic Reviews: John Wiley & Sons, Ltd, 2009.
	113. Gurusamy Kurinchi S., Kumar Y., Ramamoorthy R., Sharma D., Davidson Brian R. Vascular occlusion for elective liver resections Cochrane Database of Systematic Reviews: John Wiley & Sons, Ltd, 2009.
	114. Gurusamy Kurinchi S., Naik P., Davidson Brian R. Routine drainage for orthotopic liver transplantation Cochrane Database of Systematic Reviews: John Wiley & Sons, Ltd, 2011.
	115. Gurusamy Kurinchi S., Ramamoorthy R., Imber C., Davidson Brian R. Surgical resection versus non-surgical treatment for hepatic node positive patients with colorectal liver metastases Cochrane Database of Systematic Reviews: John Wiley & Sons, Ltd, 2010.
	116. Gurusamy Kurinchi S., Sheth H., Kumar Y., Sharma D., Davidson Brian R. Methods of vascular occlusion for elective liver resections Cochrane Database of Systematic Reviews: John Wiley & Sons, Ltd, 2009.
	117. Ha T. Y. et al. Role of hand-assisted laparoscopic surgery (HALS) for living donor right lobe harvest. HPB 16, 700-701 (2014).
	118. Hahn K. Y., Baek S. J., Joh Y. G., Kim S. H. Laparoscopic resection of transverse colon cancer: Long-term oncologic outcomes in 58 patients Journal of laparoendoscopic & advanced surgical techniques. Part A, vol. 22, 2012:561-566.
	119. Hariharan D., Constantinides V. A., Froeling F. E., Tekkis P. P., Kocher H. M. The role of laparoscopy and laparoscopic ultrasound in the preoperative staging of pancreatico-biliary cancers: a meta-analysis (Structured abstract) European Journal of Surgical Oncology, vol. 36, 2010:941-948.
	120. Hasegawa K., Kokudo N. Surgical treatment of hepatocellular carcinoma. Surgery Today 39, 833-843 (2009).
	121. Hashimoto M. et al. Evaluation of biliary abnormalities with 64-channel multidetector CT. Radiographics 28, 119-134 (2008).
	122. Hassan C. et al. Cost-effectiveness of early colonoscopy surveillance after cancer resection (Provisional abstract) Digestive and Liver Disease, vol. 41, 2009:881-885.
	123. Hegab B. et al. Day of surgery rejection of donors in living donor liver transplantation: Is it acceptable? Journal of Hepatology 54, S223 (2011).
	124. Henry David A. et al. Pre-operative autologous donation for minimising perioperative allogeneic blood transfusion Cochrane Database of Systematic Reviews: John Wiley & Sons, Ltd, 2001.
	125. Henry David A. et al. Anti-fibrinolytic use for minimising perioperative allogeneic blood transfusion Cochrane Database of Systematic Reviews: John Wiley & Sons, Ltd, 2011.
	126. Hibi T. et al. Incidence and outcomes of de novo malignancies after living donor liver transplantation. Transplantation 98, 245 (2014).
	127. Hompes D., Aerts R., Penninckx F., Topal B. Laparoscopic liver resection using radiofrequency coagulation Surgical endoscopy, vol. 21, 2007:175-180.
	128. Hünerbein M., Rau B., Schlag P. M. Laparoscopy and laparoscopic ultrasound for staging of upper gastrointestinal tumours European journal of surgical oncology, vol. 21, 1995:50-55.
	129. Hurlstone D. P. et al. EMR using dextrose solution versus sodium hyaluronate for colorectal Paris type I and 0-II lesions: a randomized endoscopist-blinded study Endoscopy, vol. 40, 2008:110-114.
	130. Iimuro Y. et al. Preceded laparoscopic splenectomy and following hepatic resection could make an alternative option for surgical treatment of hcc with severe hypersplenism and impaired liver function. Hepatology 54, 682A (2011).
	131. Iimuro Y. et al. Preceded laparoscopic splenectomy is useful for hepatic resection in HCC with severe hypersplenism and impaired liver function. HPB 14, 48 (2012).
	132. Imada R., Pacheco Junior Adhemar M., Akiba T. Endoscopic interventions for unresectable cholangiocarcinoma Cochrane Database of Systematic Reviews: John Wiley & Sons, Ltd, 2002.
	133. Iwazawa J., Ohue S., Hashimoto N., Muramoto O., Mitani T. Survival after C-arm CT-assisted chemoembolization of unresectable hepatocellular carcinoma European journal of radiology, vol. 81, 2012:3985-3992.
	134. Jahromi A. H., Shokouh-Amiri H., Jafarimehr E., Doumite D., Zibari G. B. Outcome of laparoscopic liver resection compared to open technique Hpb, vol. 14, 2012:145.
	135. Jeffery M., Hickey Brigid E., Hider Phil N. Follow-up strategies for patients treated for non-metastatic colorectal cancer Cochrane Database of Systematic Reviews: John Wiley & Sons, Ltd, 2007.
	136. Jeon H., Lee S. G. Living donor liver transplantation. Curr Opin Organ Transplant 15, 283-287 (2010).
	137. Ji W. B. et al. Robotic-assisted laparoscopic anatomic hepatectomy in China: initial experience (Provisional abstract) Annals of Surgery, vol. 253, 2011:342-348.
	138. John T. G., Greig J. D., Crosbie J. L., Miles W. F., Garden O. J. Superior staging of liver tumors with laparoscopy and laparoscopic ultrasound Annals of surgery, vol. 220, 1994:711-719.
	139. Johnson L. B., Graham J. A., Weiner D. A., Smirniotopoulos J. How does laparoscopic-assisted hepatic resection compare with the conventional open surgical approach? Journal of the American College of Surgeons 214, 717-723 (2012).
	140. Johnson N., Bryant A., Miles T., Hogberg T., Cornes P. Adjuvant chemotherapy for endometrial cancer after hysterectomy Cochrane Database of Systematic Reviews: John Wiley & Sons, Ltd, 2011.
	141. Jung D. et al. Experience of laparoscopic donor left lateral sectionectomy. Liver Transplantation 15, S139 (2009).
	142. Jung D. H. et al. Current status of LDLT in asan medical center. Liver Transplantation 19, S270 (2013).
	143. Jung D. H. et al. Current status of LDLT in ASAN medical center. HPB 16, 282-283 (2014).
	144. Kaneko H. Current status of laparoscopic liver resection. HPB 13, 171 (2011).
	145. Karner C., Chong J., Poole P. Tiotropium versus placebo for chronic obstructive pulmonary disease Cochrane Database of Systematic Reviews: John Wiley & Sons, Ltd, 2014.
	146. Katagiri H. et al. Difficulty prediction by 3D-CT for the laparoscopic-assisted hepatectomy of living donor liver transplantation. HPB 12, 422 (2010).
	147. Kayaalp C. et al. Liver transplantation from an upper midline incision. Exp Clin Transplant 9, 273-276 (2011).
	148. Khalil A. H. et al. Intraoperative 'no go' donor hepatectomy in living donor liver transplantation: A single center experience. Liver Transplantation 20, S326-S327 (2014).
	149. Kim S. E. et al. Noninvasive diagnostic criteria for hepatocellular carcinoma in hepatic masses >2cm in a hepatitis B virus-endemic area Liver international, vol. 31, 2011:1468-1476.
	150. Kim S. G. et al. Helicobacter pylori eradication on iatrogenic ulcer by endoscopic resection of gastric tumour: a prospective, randomized, placebo-controlled multi-centre trial Digestive and liver disease, vol. 45, 2013:385-389.
	151. Kim S. H., Cho S. Y., Le K. W., Park S. J., Han S. S. Upper midline incision for living donor right hepatectomy. Liver Transplantation 15, 193-198 (2009).
	152. Kinnersley P. et al. Interventions to promote informed consent for patients undergoing surgical and other invasive healthcare procedures Cochrane Database of Systematic Reviews: John Wiley & Sons, Ltd, 2013.
	153. Kitano S., Iso Y., Tomikawa M., Moriyama M., Sugimachi K. A prospective randomized trial comparing pneumoperitoneum and U-shaped retractor elevation for laparoscopic cholecystectomy Surgical endoscopy, vol. 7, 1993:311-314.
	154. Koffron A. J., Auffenberg G., Kung R., Abecassis M. Evaluation of 300 minimally invasive liver resections at a single institution: Less is more. Annals of Surgery 246, 385-392 (2007).
	155. Kokai H. et al. The new method of time-lag ligation for portosystemic shunt using coronary artery bypass graft occluder for adult living donor liver transplantation. Transplant Proc 41, 4259-4261 (2009).
	156. Kongnyuy Eugene J., Wiysonge Charles S. Interventions to reduce haemorrhage during myomectomy for fibroids Cochrane Database of Systematic Reviews: John Wiley & Sons, Ltd, 2014.
	157. Kucukmetin A., Biliatis I., Naik R., Bryant A. Laparoscopically assisted radical vaginal hysterectomy versus radical abdominal hysterectomy for the treatment of early cervical cancer Cochrane Database of Systematic Reviews: John Wiley & Sons, Ltd, 2013.
	158. Kucukmetin A., Naik R., Galaal K., Bryant A., Dickinson Heather O. Palliative surgery versus medical management for bowel obstruction in ovarian cancer Cochrane Database of Systematic Reviews: John Wiley & Sons, Ltd, 2010.
	159. Kuhry E., Schwenk W., Gaupset R., Romild U., Bonjer H. J. Long-term results of laparoscopic colorectal cancer resection Cochrane Database of Systematic Reviews: John Wiley & Sons, Ltd, 2008.
	160. Kurian M. S. et al. Hand-assisted laparoscopic donor hepatectomy for living related transplantation in the porcine model. Surgical Laparoscopy, Endoscopy and Percutaneous Techniques 12, 232-237 (2002).
	161. Kurosaki I. et al. Video-assisted living donor hemihepatectomy through a 12-cm incision for adult-to-adult liver transplantation. Surgery 139, 695-703 (2006).
	162. Lau W. Y., Lai E. C. H. Surgical practice in Hong Kong. Surgeon 4, 259-264 (2006).
	163. Lee K. W. et al. Use of an upper midline incision for living donor partial hepatectomy: A series of 143 consecutive cases. Liver Transplantation 17, 969-975 (2011).
	164. Lee S. G. et al. Current status of living donor liver transplantation in asan medical center. Transplant International 26, 295 (2013).
	165. Lee S. H. et al. Single-port laparoscopic liver surgeries. Surgical Endoscopy and Other Interventional Techniques 28, 374 (2014).
	166. Lei J., Yan L. Donor morbidity including biliary complications in living-donor liver transplantation: A single center analysis of 283 cases. Transplantation 94, e51-e52 (2012).
	167. Lennerling A. et al. Living organ donation practices in Europe. Transplantation 94, 151 (2012).
	168. Lesurtel M., Selzner M., Petrowsky H., McCormack L., Clavien P. A. How should transection of the liver be performed? A prospective randomized study in 100 consecutive patients: comparing four different transection strategies (Structured abstract) Annals of Surgery, vol. 242, 2005:814-823.
	169. Li N., Wu Y. R., Wu B., Lu M. Q. Surgical and oncologic outcomes following laparoscopic versus open liver resection for hepatocellular carcinoma: a meta-analysis (Structured abstract) Hepatology Research, vol. 42, 2012:51-59.
	170. Lin E. et al. Can current technology be integrated to facilitate laparoscopic living donor hepatectomy? Surgical Endoscopy 17, 750-753 (2003).
	171. Lin N. C., Nitta H., Wakabayashi G. Laparoscopic major hepatectomy: A systematic literature review and comparison of 3 techniques. Annals of Surgery 257, 205-213 (2013).
	172. Liu J. G., Wang Y. J., Du Z. Radiofrequency ablation in the treatment of small hepatocellular carcinoma: a meta analysis (Structured abstract) World Journal of Gastroenterology, vol. 16, 2010:3450-3456.
	173. Liu Z., Zhou Y., Zhang P., Qin H. Meta-analysis of the therapeutic effect of hepatectomy versus radiofrequency ablation for the treatment of hepatocellular carcinoma (Structured abstract) Surgical Laparoscopy, Endoscopy and Percutaneous Techniques, vol. 20, 2010:130-140.
	174. Lü M. D. et al. [Surgical resection versus percutaneous thermal ablation for early-stage hepatocellular carcinoma: a randomized clinical trial] Zhonghua yi xue za zhi, vol. 86, 2006:801-805.
	175. Luo L. X., Yu Z. Y., Bai Y. N. Laparoscopic hepatectomy for liver metastases from colorectal cancer: a meta-analysis (Provisional abstract) Journal of Laparoendoscopic and Advanced Surgical Techniques, vol. 24, 2014:213-222.
	176. Maeso S. et al. Efficacy of the Da Vinci Surgical System in abdominal surgery compared with that of laparoscopy: a systematic review and meta-analysis (Structured abstract) Annals of Surgery, vol. 252, 2010:254-262.
	177. Maguire N., Pine J., Wilson D., Agarwal A. K. Management of rectal cancer with synchronous liver metastases: A surgical dilemma Colorectal disease, vol. 16, 2014:127.
	178. Mari G. M. et al. Fast-track versus standard care in laparoscopic high anterior resection: A prospective randomized-controlled trial Surgical laparoscopy, endoscopy & percutaneous techniques, vol. 24, 2014:118-121.
	179. Marjoribanks J., Lethaby A., Farquhar C. Surgery versus medical therapy for heavy menstrual bleeding Cochrane Database of Systematic Reviews: John Wiley & Sons, Ltd, 2006.
	180. Martucci G., Burgio G., Spada M., Arcadipane A. F. Anesthetic management of totally robotic right lobe living-donor hepatectomy: new tools ask for perioperative care. Eur Rev Med Pharmacol Sci 17, 1974-1977 (2013).
	181. Marubashi S. et al. Biliary reconstruction in living donor liver transplantation: technical invention and risk factor analysis for anastomotic stricture. Transplantation 88, 1123-1130 (2009).
	182. Mathur S. et al. Randomized controlled trial of preoperative oral carbohydrate treatment in major abdominal surgery The British journal of surgery, vol. 97, 2010:485-494.
	183. McCallum I., O'Loughlin P., Robinson S., Wright Lucie J., Thomas Christophe L. G. Antibiotic-coated sutures versus non-antibiotic-coated sutures for the prevention of abdominal surgical wound infection Cochrane Database of Systematic Reviews: John Wiley & Sons, Ltd, 2014.
	184. McCarthy K., Pearson K., Fulton R., Hewitt J. Pre-operative chemoradiation for non-metastatic locally advanced rectal cancer Cochrane Database of Systematic Reviews: John Wiley & Sons, Ltd, 2012.
	185. McMahon P. M., Halpern E. F., Fernandez-del Castillo C., Clark J. W., Gazelle G. S. Pancreatic cancer: cost-effectiveness of imaging technologies for assessing resectability (Structured abstract) Radiology, vol. 221, 2001:93-106.
	186. McPhail M. J. W. et al. Laparoscopic versus open left lateral hepatectomy. Expert Review of Gastroenterology and Hepatology 3, 345-351 (2009).
	187. Medbery R. L. et al. Laparoscopic vs open right hepatectomy: a value-based analysis (Provisional abstract) Journal of the American College of Surgeons, vol. 218, 2014:929-939.
	188. Mendoza-Sanchez F. et al. Living-donor intestinal transplantation. First report in Latin-American. Revista de Investigacion Clinica 63, 96-100 (2011).
	189. Meyers W. C., Shekherdimian S., Owen S. M., Ringe B. H., Brooks A. D. Sorting through methods of dividing the liver. European Surgery - Acta Chirurgica Austriaca 36, 289-295 (2004).
	190. Milsom J. W. et al. Prospective, blinded comparison of laparoscopic ultrasonography vs. contrast-enhanced computerized tomography for liver assessment in patients undergoing colorectal carcinoma surgery Diseases of the colon and rectum, vol. 43, 2000:44-49.
	191. Mirnezami R. et al. Short- and long-term outcomes after laparoscopic and open hepatic resection: systematic review and meta-analysis (Provisional abstract) Hpb, vol. 13, 2011:295-308.
	192. Mirow L. et al. Hepatocellular carcinoma - Laparoscopic and conventional surgical treatments. Viszeralmedizin: Gastrointestinal Medicine and Surgery 25, 90-95 (2009).
	193. Miyoshi H. et al. Efficacy of prophylactic sclerotherapy in patients with hepatocellular carcinoma and varices negative for the red color sign Gastrointestinal endoscopy, vol. 45, 1997:498-502.
	194. Møller Christian H., Penninga L., Wetterslev J., Steinbrüchel Daniel A., Gluud C. Off-pump versus on-pump coronary artery bypass grafting for ischaemic heart disease Cochrane Database of Systematic Reviews: John Wiley & Sons, Ltd, 2012.
	195. Moon D. B. et al. No-touch en-bloc radical operation for unresectable hilar cholangiocarcinoma, including total hepatectomy, pancreatoduodenectoy, portal vein and hepatic artery resection, and right lobe living donor liver transplantation (video). Transplantation 94, 170 (2012).
	196. Moon D. B. et al. No-touch en-bloc radical operation for unresectable hilar cholangiocarcinoma, including total hepatectomy, pancreatoduodenectoy, portal vein and hepatic artery resection, and right lobe living donor liver transplantation. HPB 13, 93 (2011).
	197. Morino M. et al. Preoperative endoscopic sphincterotomy versus laparoendoscopic rendezvous in patients with gallbladder and bile duct stones (Structured abstract) Annals of Surgery, vol. 244, 2006:889-893.
	198. Morise Z. et al. Recent advances in the surgical treatment of hepatocellular carcinoma. World Journal of Gastroenterology 20, 14381-14392 (2014).
	199. Morrison J., Haldar K., Kehoe S., Lawrie Theresa A. Chemotherapy versus surgery for initial treatment in advanced ovarian epithelial cancer Cochrane Database of Systematic Reviews: John Wiley & Sons, Ltd, 2012.
	200. Moss Alan C., Morris E., MacMathuna P. Palliative biliary stents for obstructing pancreatic carcinoma Cochrane Database of Systematic Reviews: John Wiley & Sons, Ltd, 2006.
	201. Moulton C. et al. An Ontario Clinical Oncology Group (OCOG) randomized controlled trial (RCT) assessing FDG PET/CT in resectable liver colorectal adenocarcinoma metastases (CAM) Journal of clinical oncology, vol. 29, 2011.
	202. Mulligan D. C. Living donor safety during the performance of hepatectomy. Liver Transplantation 18, 1134-1135 (2012).
	203. Nguyen K. T., Gamblin T. C., Geller D. A. World review of laparoscopic liver resection-2,804 patients. Annals of Surgery 250, 831-841 (2009).
	204. Nguyen M. et al. Evaluation of the addition of bupivacaine to intrathecal morphine and fentanyl for postoperative pain management in laparascopic liver resection Regional anesthesia and pain medicine, vol. 35, 2010:261-266.
	205. Nishizaki T. et al. Nutritional support after hepatic resection: a randomized prospective study Hepato-gastroenterology, vol. 43, 1996:608-613.
	206. Nitta H. et al. Laparoscopic assisted major liver resection. HPB 14, 284-285 (2012).
	207. Nitta H. et al. Laparoscopy-assisted major liver resections employing a hanging technique: The original procedure. HPB 12, 308 (2010).
	208. Nitta H. et al. Laparoscopic liver resection for hepatocellular carcinoma. HPB 16, 403 (2014).
	209. Norton J. A., Doppman J. L., Jensen R. T. Curative resection in Zollinger-Ellison syndrome. Results of a 10-year prospective study Annals of surgery, vol. 215, 1992:8-18.
	210. Norum J., Olsen J. A. A cost-effectiveness approach to the Norwegian follow-up programme in colorectal cancer (Structured abstract) Annals of Oncology, vol. 8, 1997:1081-1087.
	211. O'Connor A., McNamara D., O'Moráin Colm A. Surveillance of gastric intestinal metaplasia for the prevention of gastric cancer Cochrane Database of Systematic Reviews: John Wiley & Sons, Ltd, 2013.
	212. Ohlsson B., Breland U., Ekberg H., Graffner H., Tranberg K. G. Follow-up after curative surgery for colorectal carcinoma. Randomized comparison with no follow-up Diseases of the colon and rectum, vol. 38, 1995:619-626.
	213. Oshita A. et al. Laparoscopic liver resection versus open resection for peripheral hepatocellular carcinoma in patients with chronic liver disease Surgical Endoscopy and Other Interventional Techniques, vol. 27, 2013:S35.
	214. Ova H. et al. A new technique of lateral approach for laparoscopy-assisted donor left hepatectomy preserving the caudate lobe. Liver Transplantation 17, S238 (2011).
	215. Oya H. et al. Magnetic compression anastomosis for bile duct stenosis after donor left hepatectomy: a case report. Transplant Proc 44, 806-809 (2012).
	216. Packiam V. et al. Minimally invasive liver resection: robotic versus laparoscopic left lateral sectionectomy (Provisional abstract) Journal of Gastrointestinal Surgery, vol. 16, 2012:2233-2238.
	217. Paltoglou J. P. et al. Left-sided gallbladder arising from accessory liver segment. Case report. Surgical Endoscopy and Other Interventional Techniques 25, S109 (2011).
	218. Paquet K. J., Lazar A., Rambach W., Kuhn R. [Single hepatocellular carcinoma (phi < or = 5 cm) in liver cirrhosis. Early diagnosis and surgical removal] Der Chirurg; Zeitschrift für alle Gebiete der operativen Medizen, vol. 64, 1993:784-788.
	219. Pardo F. et al. [Hepatic and pancreatic laparoscopic surgery]. An Sist Sanit Navar 28 Suppl 3, 51-59 (2005).
	220. Pfannschmidt J., Dienemann H., Hoffmann H. Surgical resection of pulmonary metastases from colorectal cancer: a systematic review of published series (Structured abstract) Annals of Thoracic Surgery, vol. 84, 2007:324-338.
	221. Pinto P. A. et al. Laparoscopic procurement model for living donor liver transplantation. Clin Transplant 17 Suppl 9, 39-43 (2003).
	222. Pinto P. A. et al. Laparoscopic procurement model for living donor liver transplantation. Clinical Transplantation 17, 39-43 (2003).
	223. Polignano F. M. et al. Laparoscopic versus open liver segmentectomy: prospective, case-matched, intention-to-treat analysis of clinical outcomes and cost effectiveness (Structured abstract) Surgical Endoscopy, vol. 22, 2008:2564-2570.
	224. Poon R. T. P. Recent advances and controversies in surgical management of liver diseases: Summary of Liver Sessions of 7th World Congress of IHPBA 2006. HPB 9, 83-86 (2007).
	225. Rahbari N. N. et al. Portal triad clamping versus vascular exclusion for vascular control during hepatic resection: a systematic review and meta-analysis (Structured abstract) Journal of Gastrointestinal Surgery, vol. 13, 2009:558-568.
	226. Rahbari N. N. et al. Meta-analysis of the clamp-crushing technique for transection of the parenchyma in elective hepatic resection: back to where we started? (Structured abstract) Annals of Surgical Oncology, vol. 16, 2009:630-639.
	227. Rahbari N. N. et al. Systematic review and meta-analysis of the effect of portal triad clamping on outcome after hepatic resection (Structured abstract) British Journal of Surgery, vol. 95, 2008:424-432.
	228. Rao A., Rao G., Ahmed I. Laparoscopic left lateral liver resection should be a standard operation (Structured abstract) Surgical Endoscopy and Other Interventional Techniques, vol. 25, 2011:1603-1610.
	229. Rao A., Rao G., Ahmed I. Laparoscopic or open liver resection? Let systematic review decide it (Structured abstract) American Journal of Surgery, vol. 204, 2012:222-231.
	230. Rao A., Rao G., Ahmed I. Laparoscopic vs. open liver resection for malignant liver disease. A systematic review (Structured abstract) Surgeon, vol. 10, 2012:194-201.
	231. Rao Ahsan M., Ahmed I. Laparoscopic versus open liver resection for benign and malignant hepatic lesions in adults Cochrane Database of Systematic Reviews: John Wiley & Sons, Ltd, 2013.
	232. Rau H. G. et al. [Liver resection with the water jet: conventional and laparoscopic surgery] Der Chirurg; Zeitschrift für alle Gebiete der operativen Medizen, vol. 67, 1996:546-551.
	233. Reddy S. K., Tsung A., Geller D. A. Laparoscopic liver resection. World journal of surgery 35, 1478-1486 (2011).
	234. Roberts I., Blackhall K., Alderson P., Bunn F., Schierhout G. Human albumin solution for resuscitation and volume expansion in critically ill patients Cochrane Database of Systematic Reviews: John Wiley & Sons, Ltd, 2011.
	235. Romanelli J. R., Kelly J. J., Litwin D. E. M. Hand-assisted laparoscopic surgery in the United States: An overview. Seminars in Laparoscopic Surgery 8, 96-103 (2001).
	236. Rowe A. J. et al. Perioperative analysis of laparoscopic versus open liver resection (Provisional abstract) Surgical Endoscopy, vol. 23, 2009:1198-1203.
	237. Rubio E. et al. Variants in visceral arterial anatomy after duodenopancreatectomy. European Journal of Surgical Oncology 38, 853 (2012).
	238. Rudow D. L. et al. Kidney and liver living donors: a comparison of experiences. Progress in transplantation (Aliso Viejo, Calif.) 15, 185-191 (2005).
	239. Rudow D. L. et al. Kidney and liver living donors: a comparison of experiences. Prog Transplant 15, 185-191 (2005).
	240. Rutten Marianne J., Leeflang Mariska M. G., Kenter Gemma G., Mol Ben Willem J., Buist M. Laparoscopy for diagnosing resectability of disease in patients with advanced ovarian cancer Cochrane Database of Systematic Reviews: John Wiley & Sons, Ltd, 2014.
	241. Saluja S. S. et al. Endoscopic or percutaneous biliary drainage for gallbladder cancer: a randomized trial and quality of life assessment Clinical gastroenterology and hepatology : the official clinical practice journal of the American Gastroenterological Association, vol. 6, 2008:944-950.e943.
	242. Satou S. et al. Initial experience of intraoperative three-dimensional navigation for liver resection using real-time virtual sonography Surgery, vol. 155, 2014:255-262.
	243. Scatton O. et al. Pure laparoscopic left lateral sectionectomy in living donors: From innovation to development in France. Annals of Surgery (2014).
	244. Schietroma M., Pessia B., Carlei F., Cecilia E. M., Amicucci G. Intestinal permeability, systemic endotoxemia, and bacterial translocation after open or laparoscopic resection for colon cancer: a prospective randomized study International journal of colorectal disease, vol. 28, 2013:1651-1660.
	245. Schmidt S., Follmann M., Malek N., Manns M. P., Greten T. F. Critical appraisal of clinical practice guidelines for diagnosis and treatment of hepatocellular carcinoma. Journal of Gastroenterology and Hepatology (Australia) 26, 1779-1786 (2011).
	246. Schmidt-Hansen M. et al. PET-CT for assessing mediastinal lymph node involvement in patients with suspected resectable non-small cell lung cancer Cochrane Database of Systematic Reviews: John Wiley & Sons, Ltd, 2014.
	247. Schoemaker D., Black R., Giles L., Toouli J. Yearly colonoscopy, liver CT, and chest radiography do not influence 5-year survival of colorectal cancer patients Gastroenterology, vol. 114, 1998:7-14.
	248. Schoppmeyer K., Weis S., Mössner J., Fleig Wolfgang E. Percutanous ethanol injection or percutaneous acetic acid injection for early hepatocellular carcinoma Cochrane Database of Systematic Reviews: John Wiley & Sons, Ltd, 2009.
	249. Schultz J. F., Bell J. D., Goldstein R. M., Kuhn J. A., McCarty T. M. Hepatic tumour imaging using iron oxide MRI: comparison with computed tomography, clinical impact, and cost analysis (Structured abstract) Annals of Surgical Oncology, vol. 6, 1999:691-698.
	250. Schwenk W., Haase O., Neudecker Jens J., Müller Joachim M. Short term benefits for laparoscopic colorectal resection Cochrane Database of Systematic Reviews: John Wiley & Sons, Ltd, 2005.
	251. Sekimoto T. et al. Virtual laparoscopy: Initial experience with three-dimensional ultrasonography to characterize hepatic surface features European journal of radiology, vol. 82, 2013:929-934.
	252. Shchepotin I. B., Chorny V., Hanfelt J., Evans S. R. Palliative superselective intra-arterial chemotherapy for advanced nonresectable gastric cancer Journal of gastrointestinal surgery, vol. 3, 1999:426-431.
	253. Shen L. et al. Interventions for non-tubal ectopic pregnancy Cochrane Database of Systematic Reviews: John Wiley & Sons, Ltd, 2014.
	254. Shen Z. The Liver Transplant Program at Tianjin First Center Hospital. Clinical transplants, 203-211 (2011).
	255. Shimada M. et al. Laparoscopic hepatectomy for hepatocellular carcinoma Surgical endoscopy, vol. 15, 2001:541-544.
	256. Shimada M. et al. Advances in the liver cancer surgery. Hepatology International 4, 48 (2010).
	257. Shin M., Kwon C. H. D., Kim S. J., Lee S. K., Joh J. W. The unreachable goal in living-donor hepatectomy: Zero percent donor morbidity. Transplantation 94, e52-e53 (2012).
	258. Shinoda M. et al. Left-side hepatectomy in living donors: Through a reduced upper-midline incision for liver transplantation. Transplantation Proceedings 46, 1400-1406 (2014).
	259. Shinoda M. et al. Attempts to reduce incision length in living donor lateral segmentectomy and left hepatectomy for living donor liver transplantation. American Journal of Transplantation 12, 357 (2012).
	260. Shoukry Hafez A. M. Laparoscopic hepatectomy, analysis of the safety, feasibility and reproducibility in a preliminary study Surgical Endoscopy and Other Interventional Techniques, vol. 28, 2014:S136.
	261. Showell Marian G., Brown J., Clarke J., Hart Roger J. Antioxidants for female subfertility Cochrane Database of Systematic Reviews: John Wiley & Sons, Ltd, 2013.
	262. Simillis C. et al. Laparoscopic versus open hepatic resections for benign and malignant neoplasms: a meta-analysis (Structured abstract) Surgery, vol. 141, 2007:203-211.
	263. Simpson E. et al. Recombinant factor VIIa for the prevention and treatment of bleeding in patients without haemophilia Cochrane Database of Systematic Reviews: John Wiley & Sons, Ltd, 2012.
	264. Singh H. et al. Propofol for sedation during colonoscopy Cochrane Database of Systematic Reviews: John Wiley & Sons, Ltd, 2008.
	265. Singh M. et al. Midline mini-laparotomy for living donor hepatectomy. American Journal of Transplantation 12, 439 (2012).
	266. Sladden Michael J. et al. Surgical excision margins for primary cutaneous melanoma Cochrane Database of Systematic Reviews: John Wiley & Sons, Ltd, 2009.
	267. Smith Mark D. et al. Preoperative carbohydrate treatment for enhancing recovery after elective surgery Cochrane Database of Systematic Reviews: John Wiley & Sons, Ltd, 2014.
	268. Song G. W., Lee S. G. Living donor liver transplantation. Current Opinion in Organ Transplantation 19, 217-222 (2014).
	269. Song H., Lu D., Navaratnam K., Shi G. Aromatase inhibitors for uterine fibroids Cochrane Database of Systematic Reviews: John Wiley & Sons, Ltd, 2013.
	270. Sotiropoulos G. C. et al. Surgery for hepatocellular carcinoma arising in hereditary hemochromatosis. European Surgical Research 38, 371-376 (2006).
	271. Soyama A. et al. A hybrid method of laparoscopic-assisted open liver resection through a short upper midline laparotomy can be applied for all types of hepatectomies. Surgical Endoscopy and Other Interventional Techniques 28, 203-211 (2014).
	272. Soyama A. et al. Standardized less invasive living donor hemihepatectomy using the hybrid method through a short upper midline incision. Transplant Proc 44, 353-355 (2012).
	273. Spada M. et al. First european report of an entirely robotic right hepatectomy for adult living related liver transplantation. Transplant International 26, 55 (2013).
	274. Stang A., Fischbach R., Teichmann W., Bokemeyer C., Braumann D. A systematic review on the clinical benefit and role of radiofrequency ablation as treatment of colorectal liver metastases (Structured abstract) European Journal of Cancer, vol. 45, 2009:1748-1756.
	275. Stones W., Cheong Ying C., Howard Fred M., Singh S. Interventions for treating chronic pelvic pain in women Cochrane Database of Systematic Reviews: John Wiley & Sons, Ltd, 2005.
	276. Strotzer M. et al. Diagnosis of liver metastases from colorectal adenocarcinoma. Comparison of spiral-CTAP combined with intravenous contrast-enhanced spiral-CT and SPIO-enhanced MR combined with plain MR imaging Acta radiologica (Stockholm, Sweden : 1987), vol. 38, 1997:986-992.
	277. Suh K. S. et al. Transverse incisional donor right hepatectomy assisted by laparoscopy. HPB 16, 723 (2014).
	278. Suh K. S. et al. Laparoscopy-assisted donor right hepatectomy using a hand port system preserving the middle hepatic vein branches. World Journal of Surgery 33, 526-533 (2009).
	279. Suh S. W. et al. Comparision of patient satisfaction according to different incision methods for donor hepatectomy in living donor liver transplantation. Liver Transplantation 20, S253-S254 (2014).
	280. Suo T. et al. Oral traditional Chinese medication for adhesive small bowel obstruction Cochrane Database of Systematic Reviews: John Wiley & Sons, Ltd, 2012.
	281. Tabrizian P., Schwartz M. E. Surgical management of hepatocellular carcinoma. Mount Sinai Journal of Medicine 79, 223-231 (2012).
	282. Tacklind J., Fink Howard A., MacDonald R., Rutks I., Wilt Timothy J. Finasteride for benign prostatic hyperplasia Cochrane Database of Systematic Reviews: John Wiley & Sons, Ltd, 2010.
	283. Tai Q. W., Zhang J. H., Wen H. Short-term effectiveness and safety of laparoscopic versus open left hepatectomy for primary cancer: a systematic review (Provisional abstract) Chinese Journal of Evidence-Based Medicine, vol. 13, 2013:26-30.
	284. Takahara T., Wakabayashi G., Hasegawa Y., Nitta H. Minimally invasive donor hepatectomy: Evolution from hybrid to pure laparoscopic techniques. Annals of Surgery (2014).
	285. Tan H. P. et al. Alemtuzumab pretreatment and tacrolimus monotherapy in living-donor liver and kidney transplantation. Expert Review of Pharmacoeconomics and Outcomes Research 7, 113-118 (2007).
	286. Tang T., Lord Jonathan M., Norman Robert J., Yasmin E., Balen Adam H. Insulin-sensitising drugs (metformin, rosiglitazone, pioglitazone, D-chiro-inositol) for women with polycystic ovary syndrome, oligo amenorrhoea and subfertility Cochrane Database of Systematic Reviews: John Wiley & Sons, Ltd, 2012.
	287. Tanner Karuna S., Thirlby R., Biehl T., Veenstra D. Cost-effectiveness of laparoscopy versus laparotomy for initial surgical evaluation and treatment of potentially resectable hepatic colorectal metastases: a decision analysis (Structured abstract) Journal of Surgical Oncology, vol. 97, 2008:396-403.
	288. Tarantino I. et al. Transvaginal rigid-hybrid natural orifice transluminal endoscopic surgery technique for anterior resection treatment of diverticulitis: A feasibility study Surgical Endoscopy and Other Interventional Techniques, vol. 25, 2011:3034-3042.
	289. Thudi K. R. et al. Cat scratch disease causing hepatic masses after liver transplant. Liver Int 27, 145-148 (2007).
	290. Tiong L., Maddern G. J. Systematic review and meta-analysis of survival and disease recurrence after radiofrequency ablation for hepatocellular carcinoma (Structured abstract) British Journal of Surgery, vol. 98, 2011:1210-1224.
	291. Topfer L. A. Water jet systems for surgical dissection and resection (Structured abstract) Health Technology Assessment Database, 2004.
	292. Traut U. et al. Systemic prokinetic pharmacologic treatment for postoperative adynamic ileus following abdominal surgery in adults Cochrane Database of Systematic Reviews: John Wiley & Sons, Ltd, 2008.
	293. Troisi R. I. et al. Evolution of laparoscopic left lateral sectionectomy without the Pringle maneuver: Through resection of benign and malignant tumors to living liver donation. Surgical Endoscopy and Other Interventional Techniques 25, 79-87 (2011).
	294. Troisi R. I. et al. Pure laparoscopic full-left living donor hepatectomy for calculated small-for-size LDLT in adults: Proof of concept. American Journal of Transplantation 13, 2472-2478 (2013).
	295. Tsao May N. et al. Whole brain radiotherapy for the treatment of newly diagnosed multiple brain metastases Cochrane Database of Systematic Reviews: John Wiley & Sons, Ltd, 2012.
	296. Tsukamoto T., Kanazawa A., Kodai S., Kubo S. Recent progress in laparoscopic liver resection. Clinical Journal of Gastroenterology 6, 8-15 (2013).
	297. Tuttle-Newhall J. E. In brief. Current Problems in Surgery 42, 139-141 (2005).
	298. Tzortzopoulou A. et al. Single dose intravenous propacetamol or intravenous paracetamol for postoperative pain Cochrane Database of Systematic Reviews: John Wiley & Sons, Ltd, 2011.
	299. Vanounou T. et al. Comparing the clinical and economic impact of laparoscopic versus open liver resection (Provisional abstract) Annals of Surgical Oncology, vol. 17, 2010:998-1009.
	300. Vennix S. et al. Laparoscopic versus open total mesorectal excision for rectal cancer Cochrane Database of Systematic Reviews: John Wiley & Sons, Ltd, 2014.
	301. Verma R., Nelson Richard L. Prophylactic nasogastric decompression after abdominal surgery Cochrane Database of Systematic Reviews: John Wiley & Sons, Ltd, 2007.
	302. Vettoretto N. et al. Laparoscopic-endoscopic rendezvous versus preoperative endoscopic sphincterotomy for common bile duct stones in patients undergoing laparoscopic cholecystectomy Cochrane Database of Systematic Reviews: John Wiley & Sons, Ltd, 2013.
	303. Voigt R., Settmacher U. Current aspects in the curative surgical treatment of primary liver tumors. Verdauungskrankheiten 29, S242-S251 (2011).
	304. Wacha H. Surgical and endoscopic procedures - Risk-adapted antibiotic prophylaxis in surgery. [German] Klinikarzt, vol. 31, 2002:299-303.
	305. Wakabayashi G. Laparoscopic anatomical liver resection for hepatocellular carcinoma. HPB 13, 26-27 (2011).
	306. Wakabayashi G. Laparoscopic anatomical resection for HCC: Indication and technique consideration. Hepatology International 6, 18 (2012).
	307. Wakabayashi G. et al. Standardization of basic skills for laparoscopic liver surgery towards laparoscopic donor hepatectomy. Journal of Hepato-Biliary-Pancreatic Surgery 16, 439-444 (2009).
	308. Walker C. B. et al. Minimal modulation of lymphocyte and natural killer cell subsets following minimal access surgery American journal of surgery, vol. 177, 1999:48-54.
	309. Wang P., Wang H. W., Zhong T. D. Influence of different anesthesia on liver and renal function in elderly patients undergoing laparoscopic colon or rectal resection Hepato-gastroenterology, vol. 60, 2013:79-82.
	310. Wang X. et al. Validation of the laparoscopically stapled approach as a standard technique for left lateral segment liver resection (Provisional abstract) World Journal of Surgery, vol. 37, 2013:806-811.
	311. Wei M. et al. Laparoscopic versus open hepatectomy with or without synchronous colectomy for colorectal liver metastasis: a meta-analysis (Provisional abstract) Plos One, vol. 9, 2014:e87461.
	312. Wei X., Chen Z., Yang X., Wu T. Chinese herbal medicines for esophageal cancer Cochrane Database of Systematic Reviews: John Wiley & Sons, Ltd, 2009.
	313. Weis S., Franke A., Mössner J., Jakobsen Janus C., Schoppmeyer K. Radiofrequency (thermal) ablation versus no intervention or other interventions for hepatocellular carcinoma Cochrane Database of Systematic Reviews: John Wiley & Sons, Ltd, 2013.
	314. Weng M. et al. Radiofrequency ablation versus resection for colorectal cancer liver metastases: a meta-analysis (Structured abstract) Plos One, vol. 7, 2012:e45493.
	315. Wereldsma J. C., Bruggink E. D., Meijer W. S., Roukema J. A., Putten W. L. Adjuvant portal liver infusion in colorectal cancer with 5-fluorouracil/heparin versus urokinase versus control. Results of a prospective randomized clinical trial (colorectal adenocarcinoma trial I) Cancer, vol. 65, 1990:425-432.
	316. Wigham A., Alexander Grant L. Radiologic Assessment of Hepatobiliary Surgical Complications. Seminars in Ultrasound, CT and MRI 34, 18-31 (2013).
	317. Wileman Samantha M., McCann S., Grant Adrian M., Krukowski Zygmunt H., Bruce J. Medical versus surgical management for gastro-oesophageal reflux disease (GORD) in adults Cochrane Database of Systematic Reviews: John Wiley & Sons, Ltd, 2010.
	318. Wilson Colin H., Sanni A., Rix David A., Soomro Naeem A. Laparoscopic versus open nephrectomy for live kidney donors Cochrane Database of Systematic Reviews: John Wiley & Sons, Ltd, 2011.
	319. Wong Rebecca K. S., Tandan V., De Silva S., Figueredo A. Pre-operative radiotherapy and curative surgery for the management of localized rectal carcinoma Cochrane Database of Systematic Reviews: John Wiley & Sons, Ltd, 2007.
	320. Wong-Lun-Hing E. et al. The orange II plus-trial: An international multicentre randomised controlled trial of open versus laparoscopic hemihepatectomie Hpb, vol. 14, 2012:425.
	321. Wong-Lun-Hing E. M. et al. A survey in the hepatopancreatobiliary community on ways to enhance patient recovery. HPB 14, 818-827 (2012).
	322. Worthington Helen V. et al. Interventions for preventing oral mucositis for patients with cancer receiving treatment Cochrane Database of Systematic Reviews: John Wiley & Sons, Ltd, 2011.
	323. Wu Y. M., Hu R. H. Robotic right lobectomy for living donor of liver. Liver Transplantation 20, S120 (2014).
	324. Xiong J. J. et al. Meta-analysis of laparoscopic vs open liver resection for hepatocellular carcinoma (Provisional abstract) World Journal of Gastroenterology, vol. 18, 2012:6657-6668.
	325. Yamamoto S. et al. Short-term surgical outcomes from a randomized controlled trial to evaluate laparoscopic and open D3 dissection for stage II/III colon cancer: Japan clinical oncology group study JCOG 0404 Annals of surgery, vol. 260, 2014:23-30.
	326. Yao F. Y. et al. Excellent outcome following down-staging of hepatocellular carcinoma prior to liver transplantation: An intention-to-treat analysis. Hepatology 48, 819-827 (2008).
	327. Yao F. Y. et al. Excellent long-term outcome following down-staging of hepatocellular carcinoma prior to liver transplantation: an intention-to-treat analysis Hepatology (Baltimore, Md.), vol. 46, 2007:309a.
	328. Ye B. D. et al. Omeprazole may be superior to famotidine in the management of iatrogenic ulcer after endoscopic mucosal resection: a prospective randomized controlled trial Alimentary pharmacology & therapeutics, vol. 24, 2006:837-843.
	329. Yegiants A. et al. Effect of fully laparoscopic left hepatectomy on donor interest in living donor liver transplantation (LDLT). Hepatology 60, 511A (2014).
	330. Yiannakopoulou E. C., Nikiteas N., Perrea D., Tsigris C. Characterization of neuroendocrine stress response to minimally invasive surgery-systematic review of animal data. Surgical Endoscopy and Other Interventional Techniques 25, S44 (2011).
	331. Yin W., Chen J., Lee C., Chang C. Laparoscopic surgery in transplanted patients: A series of case report and literature review. Transplantation 98, 663 (2014).
	332. Yin Z., Fan X., Ye H., Yin D., Wang J. Short- and long-term outcomes after laparoscopic and open hepatectomy for hepatocellular carcinoma: a global systematic review and meta-analysis (Provisional abstract) Annals of Surgical Oncology, vol. 20, 2013:1203-1215.
	333. You Y. K. et al. SIngle-port laparoscopic liver surgeries: The initial experiences in 89 cases. HPB 13, 19 (2011).
	334. You Y. K. et al. Single-port laparoscopic liver surgery-170 cases review. Surgical Endoscopy and Other Interventional Techniques 28, S17 (2014).
	335. Zaydfudim V., Wright J. K., Pinson C. W. Liver transplantation for iatrogenic porta hepatis transection. American Surgeon 75, 313-316 (2009).
	336. Zerbi A. et al. Surgical treatment of pancreatic endocrine tumours in Italy: Results of a prospective multicentre study of 262 cases Langenbeck's archives of surgery / Deutsche Gesellschaft fur Chirurgie, vol. 396, 2011:313-321.
	337. Zhang J. et al. Chinese herbal medicine for subfertile women with polycystic ovarian syndrome Cochrane Database of Systematic Reviews: John Wiley & Sons, Ltd, 2010.
	338. Zhao X. X. et al. Safety and effectiveness of radiofrequency combined with laparoscopic cholecystectomy in management of liver cancer near the gallbladder World Chinese Journal of Digestology, vol. 21, 2013:2212-2216.
	339. Zhou Y., Xiao Y., Wu L., Li B., Li H. Laparoscopic liver resection as a safe and efficacious alternative to open resection for colorectal liver metastasis: a meta-analysis (Provisional abstract) BMC Surgery, vol. 13, 2013:44.
	340. Zhou Y. et al. Meta-analysis of radiofrequency ablation versus hepatic resection for small hepatocellular carcinoma (Structured abstract) BMC Gastroenterology, vol. 10:78, 2010.
	341. Zhou Y. M., Shao W. Y., Zhao Y. F., Xu D. H., Li B. Meta-analysis of laparoscopic versus open resection for hepatocellular carcinoma (Provisional abstract) Digestive Diseases and Sciences, vol. 56, 2011:1937-1943.
